# A general end-to-end diagnosis framework for manufacturing systems

**DOI:** 10.1093/nsr/nwz190

**Published:** 2019-11-21

**Authors:** Ye Yuan, Guijun Ma, Cheng Cheng, Beitong Zhou, Huan Zhao, Hai-Tao Zhang, Han Ding

**Affiliations:** 1 School of Artificial Intelligence and Automation, MOE Key Lab of Intelligent Control and Image Processing, Huazhong University of Science and Technology, Wuhan 430074, China; 2 State Key Laboratory of Digital Manufacturing Equipment and Technology, Huazhong University of Science and Technology, Wuhan 430074, China; 3 School of Mechanical Science and Engineering, Huazhong University of Science and Technology, Wuhan 430074, China

**Keywords:** manufacturing systems, deep learning, diagnosis and monitoring

## Abstract

The manufacturing sector is envisioned to be heavily influenced by artificial-intelligence-based technologies with the extraordinary increases in computational power and data volumes. A central challenge in the manufacturing sector lies in the requirement of a general framework to ensure satisfied diagnosis and monitoring performances in different manufacturing applications. Here, we propose a general data-driven, end-to-end framework for the monitoring of manufacturing systems. This framework, derived from deep-learning techniques, evaluates fused sensory measurements to detect and even predict faults and wearing conditions. This work exploits the predictive power of deep learning to automatically extract hidden degradation features from noisy, time-course data. We have experimented the proposed framework on 10 representative data sets drawn from a wide variety of manufacturing applications. Results reveal that the framework performs well in examined benchmark applications and can be applied in diverse contexts, indicating its potential use as a critical cornerstone in smart manufacturing.

## INTRODUCTION

In recent decades, it has been envisioned that sensory data measured in manufacturing processes, including vibration, pressure, temperature and energy data, can be used as features for artificial-intelligence (AI) algorithms [1–3]. AI algorithms have the potential to localize faults or even predict faults before they occur. In this way, run-to-failure maintenance could be replaced by condition-based or predictive maintenance that would be more effective in reducing unnecessary maintenance cost while guaranteeing the reliability of the machinery [4]. However, the existing diagnosis and monitoring techniques most focus on specific tasks; advanced approaches should be developed to form a general framework to produce satisfied performances after simple tuning of parameters in different manufacturing applications.

Model-based and data-driven approaches are two main techniques for diagnosis and monitoring. Model-based approaches for fault monitoring use mathematical models to provide insights into the failure mechanism of mechanical systems [5,6]. Faults are diagnosed by monitoring discrepancies between model predictions and the actual measurements. With the increasing volume of data captured from sensors during manufacturing processes, data-driven approaches have been gaining considerable attention [7,8]. Data-driven approaches are featured by building models without using the knowledge of the failure mechanism, but can perform excellent prediction results [9,10]. The measured sensory signals have often been processed via feature extraction [11] to represent the complete signals manually. The extracted features are then used to train the system using standard classification and regression methods to allow predictions to be made in a case-by-case manner [12–14]. However, both model-based and data-driven approaches are highly tuned to applications and could not be generalized to other applications without substantial efforts. Consequently, there exists an urgent need for a method that can simultaneously provide convenience for feature extraction and offer universality for use in diverse manufacturing applications.

On the other hand, the Convolutional Neural Network (CNN) [15], as an important type of deep learning, obtained remarkable results in ImageNet in 2012 [16] and has gradually become a representative method that is used in medical-diagnosis [17], image-recognition [18] and speech-recognition [19] applications. When compared with other machine-learning algorithms, the advantage of CNN is that it enables automatic feature extraction from raw data and can thus eliminate any dependence on prior knowledge [20], which brings inspiration that CNN could provide unified, end-to-end solutions to industrial problems.

This paper transforms manufacturing-monitoring problems into a unified supervised-learning framework. In particular, it proposes a general end-to-end framework, i.e. a CNN that can extract features automatically and solve the problems accurately. Its outperformance is verified via using 10 measurement data sets for different manufacturing problems. Two open benchmark data sets including Case Western Reserve University's bearing data [21] and hydraulic-system data [22], five experiment data sets performed in the lab including airplane-girder simulation-damage data [23], broken-tool data, the bearing data [23], tool-wear data and gearbox data [24] were all converted into classification problems. Moreover, National Aeronautics and Space Administration (NASA) tool-wearing data [25], battery data [26] and the Center of Advanced Life Cycle Engineering (CALCE) battery data [27] were converted into regression problems. Higher than 95% accuracies are achieved using a unified CNN framework for manufacturing diagnosis problems, while small monitoring errors are achieved for condition monitoring problems, indicating the proposed framework has a good application prospect in the manufacturing field. In addition, the robustness of the proposed framework is investigated by adding different levels of additive noises to the raw signals in diagnosis tasks.

## RESULTS

Rolling bearing fault detection and classification are used here as an illustrative example for the proposed framework; other applications can be found in the Supplementary Data. Rolling bearings are vital components in many types of rotating machinery, ranging from simple electrical fans to complex machine tools. More than half of machinery defects are generally related to bearing faults. Typically, a rolling bearing fault can lead to machine shutdown, chain damage and even human casualties [28]. Bearing vibration fault signals are usually caused by localized defects in three components: the rolling elements, the outer race and the inner race. When bearings degrade near the end of their lifetimes, instances of deformation, cracking and burning among these components may cause spindle deviation and further serious damage to mechanical systems.

**Figure 1.**
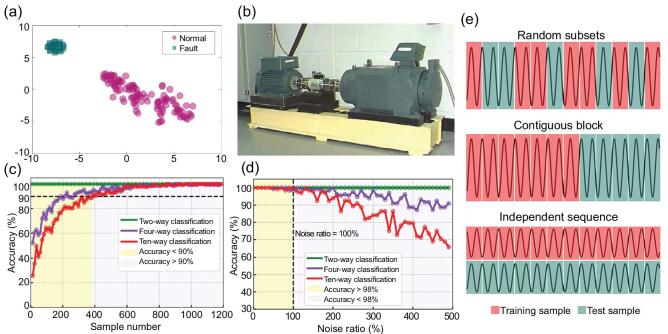
Classification results on the CWRU-bearing-test data set, visualized in a 2D feature space. (a) t-SNE visualization of the binary-classification task (normal and fault). (b) The experiment platform in the CWRU-bearing data center. (c) Evolution curves of the accuracy variations regarding the two-way (binary), four-way and ten-way classification tasks, where the sample number is increased from 10 to 1200. (d) The curves of accuracies with the noise ratio vary from 0% to 500% for the three classification models, where the accuracies all surpass 98% when the noise ratio is less than or equal to 100%. (e) Schematic diagram of the three cross-validation methods (random subsets, contiguous block and independent sequence).

A bearing data set provided by the Case Western Reserve University (CWRU) data center—which is regarded as a benchmark for the bearing fault diagnosis problem—is used to validate the effectiveness of our proposed framework. An experimental platform (illustrated in Fig. 1b [29]) was used to conduct the signals to be used for defect detection on bearings with three different fault diameters (7, 14 and 21 mils (1 mil = 0.001 inches)). Vibration signals in different conditions from the inner race, the outer race and the rolling elements for all fault diameters were acquired using accelerometers. The data set originally consisted of four rotating speeds (1797, 1772, 1750 and 1730 rpm) and in total had 4 normal samples and 52 faulty samples. We formulate this as a fault-diagnosis problem by classifying the fault types as representations of the following three problems: (i) binary classification (normal plus faulty conditions), (ii) four-way classification (normal plus three main faulty conditions with different rotating speeds) and (iii) ten-way classification (normal plus three main faulty conditions for each of the faulty diameters).

**Figure 2.**
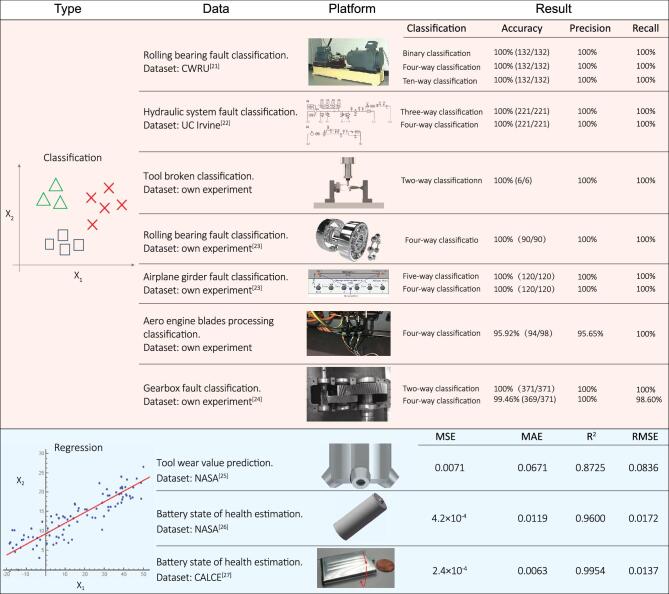
Summary of the classification and regression results of different data sets. The data sets for classification problems include: CWRU-bearing data; hydraulic-system data; tool-broken data; bearing data; airplane-girder data; blades-processing data; gearbox data. The data sets for the supervised-regression problems include: NASA tool-wear data; NASA battery data; CALCE data. Specifically, for the multi-classification problem, we define the first class as the positive class to calculate the precision, recall and accuracy according to Equations (1–3).

Each sample in the original data set contains a different number of time-course measurements. To increase the number of samples for training a more accurate model, we reshape the samples here to ensure that each sample has 6000 time-course measurements consistently. In total, 1320 samples are reconstructed from the original data set. Considering that the potential time dependency existed among the reconstructed samples, we apply three standard cross-validation methods (random subsets, contiguous block and independent sequence [30], which are depicted in Fig. 1e) to evaluate the performance of the CNN method. For the random-subsets method, the entire pre-processed data set is constructed and then randomly divided into 90% for training (1188 samples) and 10% for test (132 samples). Figure 1a presents the t-SNE visualization [31] of the binary-classification features before the final classifier. Features for normal and faulty signals are clearly separated into two clusters indicating that a good classification can be easily obtained by selecting a proper final classifier. Figure 1c demonstrates that the classification accuracy will be improved with the increasing of the sample number. We also reveal that more samples are required to obtain a promising result for a more complex problem, such as the ten-way classification task that requires at least 400 samples for training a model with 90% accuracy. Classification results and evaluation metrics are summarized in Fig. 2, where all three models achieve 100% (i.e. 132 of 132 test samples) fault classification, and are consistent over different randomization. For the contiguous-block method, we divide the 1320 samples into a training set and a test set according to time evolution, and the proportion of the test set varied from 10% to 50% of the entire record time of the original sample; the accuracies are greater than 95% for all experiments using the contiguous-block method. For the independent-sequence method, to eliminate the confused dependency, we divide the data set into a completely independent training set and a test set, i.e. the training set and test set correspond to different rotating speeds, where data with rotating speeds of 1797, 1750 and 1730 rpm are used for training and data with rotating speeds of 1772 rpm are used for test. Similar results are obtained using the independent-sequence method: 100% (340/340), 100% (340/340) and 98.82% (336/400) for two-way classification, four-way classification and ten-way classification, respectively. The experiment results of three cross-validation methods are summarized in Table 1.

**Table 1.** Three cross-validation methods (random subsets, contiguous block, independent sequence) are employed to verify the effectiveness of the model in CWRU data; 10%, 20%, 30%, 40% and 50% test schemes for the contiguous-block method are used. X% means that the first (100–X)% of the total data set is used for training and the rest (X% of the data) is used for test.

			Contiguous block				
	Random	Independent					
	subsets	sequence	10% test	20% test	30% test	40% test	50% test
**Two-way**	100% (132/132)	100% (340/340)	100% (132/132)	100% (262/264)	100% (396/396)	100% (528/528)	100% (660/660)
**Four-way**	100% (132/132)	100% (340/340)	100% (132/132)	99.24% (262/264)	97.22% (385/396)	95.83% (506/528)	96.21% (635/660)
**Ten-way**	100% (132/132)	98.82% (336/340)	99.24% (131/132)	98.86% (261/264)	98.48% (390/396)	96.21% (508/528)	96.67% (638/660)

For further evaluation of the classification results, we used the following three assessment metrics that are commonly used in machine learning to evaluate the classification performance with test data using the random-subsets method: (i) precision, (ii) recall and (iii) accuracy, which are defined as follows:

(1) \begin{equation*}{\textrm {precision}}\ = \frac{{\rm TP}}{{\rm TP + FP}}{\rm{\ }} \times {\rm{\ }}100{\rm{\% }},\end{equation*}

(2) \begin{equation*} {\rm{recall\ }} = \frac{{{\rm{TP}}}}{{{\rm{TP}} + {\rm{FN}}}}{\rm{\ \ }} \times {\rm{\ }}100{\rm{\% }}, \end{equation*}

(3) \begin{equation*} \rm{accuracy} = \frac{{{\rm{TP}} + {\rm{TN}}}}{{{\rm{TP}} + {\rm{TN}} + {\rm{FP}} + {\rm{FN}}}}{\rm{\ \ }} \times \ 100\% , \end{equation*}

where the abbreviations TP, FP, FN and TN denote the numbers of true positives, false positives, false negatives and true negatives, respectively [32–34]. In our four-way and ten-way classification in random subsets, we regarded the first class as the positive class while others are negative classes for computing these metrics. Across the three classification tests, the defined assessment metrics all achieved results of 100%.

These results demonstrate that, without prior knowledge (manufacturing parameters and failure mechanism), measurement data as well as their labels suffice to classify fault types accurately and thereby pinpoint the location of faults, which makes the fixing process efficient. In addition, the proposed framework requires an average of 5 min for training using a standard GTX 1080 GPU. With the obtained trained model, the CNN performs fault-prediction results within 0.05 s on the same GPU, which is fast enough compared to the sampling time of 0.5 s. Therefore, the proposed algorithm can be implemented online to localize faults in real time.

### Generalizability of the proposed CNN framework

**Figure 3.**
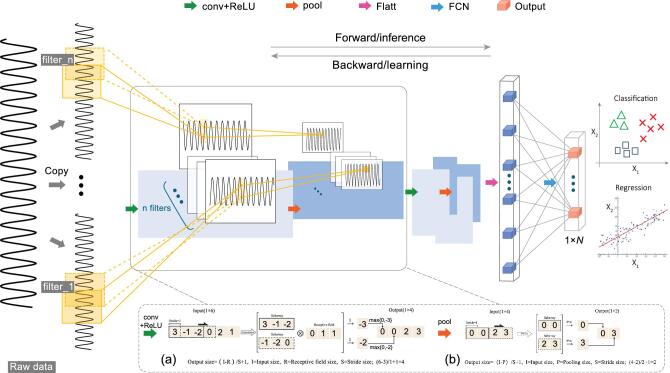
An illustration of a CNN model for a classification/regression task. This framework is a fully automated system. Raw data are generated by the manufacturing system and processed, and they go through the CNN operations. The input raw data are passed through convolutional layers (a), Max-pooling layers (b) and fully connected layers, as explained in the ‘Convolutional neural networks’ section. A flattening operation is employed before the data are fed into the first fully connected layer. The output layer with 1${\rm{\ }} \times N\ $size resulting (*N* is an integer for classification categorizes or equals 1 for regression) from the CNN model can be fed back to the manufacturing system for decision-making.

A major feature of the proposed framework can also be generalized for a wide range of other applications with high metrics, including greater accuracy, precision and recall (summarized in Fig. 2). Here, we focus on two other representative applications using the proposed CNN framework (Fig. 3):

- Hydraulic-system-condition classification: with its excellent performance in creating movement or repetition [35,36], hydraulic-system-based equipment has been widely used in many applications, including manufacturing, robotics and steel processing. However, the fluid in a hydraulic system is highly pressurized, extremely hot and even toxic, which bring a high level of hazards to the workers and the surrounding environment. Our CNN fault-prediction algorithm for hydraulic systems can generate hazard-warning signals to prevent chemical burns to the workers, igniting nearby materials and causing explosions in real time. Hydraulic-system-condition monitoring is a classification task. We chose CNN as the base model to make predictions for different conditions. Four condition classifications corresponding to different hazard types and levels are conducted: (a) a three-way classification for cooler condition, (b) a four-way classification for valve condition, (c) a three-way classification model for internal pump leakage and (d) a four-way classification model for the hydraulic accumulator. The algorithm achieved accuracies of 100% in both cooler-condition and valve-condition classifications. Meanwhile, the pump-leakage and hydraulic-accumulator classifications also achieved satisfactory accuracies, at 98.19% and 99.10%, respectively.
- NASA lithium-ion battery data for State of Health (SOH) estimation: lithium-ion batteries (LiBs) are the auxiliary or main power sources for many electronic systems, including medical devices, aerospace systems, smartphones and electric vehicles [37]. Estimating the SOH is the key issue for evaluating the health status of LiBs. A benchmark of industrial lithium-ion battery data obtained by NASA is used to estimate battery SOH. CNN models are trained for this data set and the smallest average RMSE (Equation (9) in the Supplementary Data) value of 0.0172 mm is achieved with respect to the smallest error of 0.0264 mm that has been achieved in previous related work [38]. Detailed descriptions of the data structures and the established models for these applications and several further, diverse cases can be found in the Supplementary Data.

### Interpretability of the proposed CNN framework

To validate the generality of the proposed CNN-algorithm framework for fault prediction, it is key to understand how CNN extracts meaningful features from the manufacturing data. However, interpreting deep neural networks remains a notoriously difficult task in the literature. Inspired by practical successful studies in medicine [39] and biology [40], we have developed a method for manufacturing data to visualize the general features, such as frequency, phase or amplitude, extracted by a CNN model that contribute to the fault prediction. Note that, in this study, we only focus on revealing the relationship between the convolutional layers (outputs before fully connected layers) and the hidden features in the manufacturing data.

**Figure 4.**
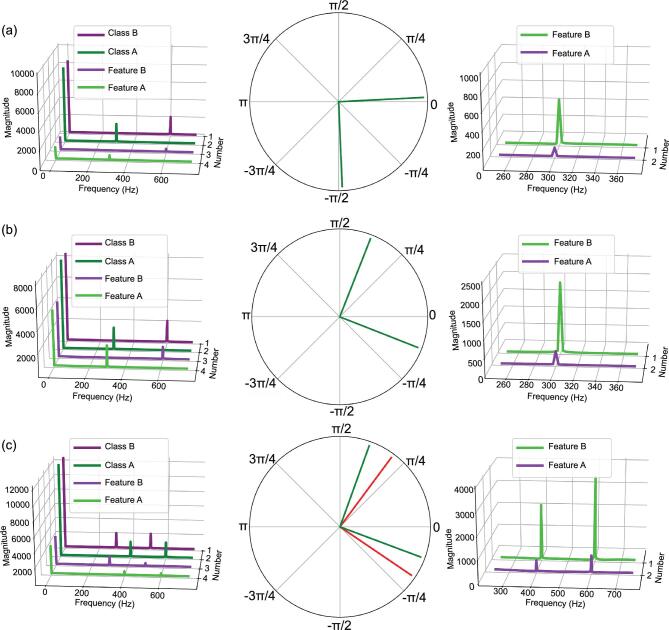
Interpretation of the CNN model for manufacturing data. Two classes (class A and class B) of time-series signals with different coefficient settings (${F_n},\ \ {\phi _n},\ {a_n},\ {\rm and}\ u$ in Equation (13)) are formulated to represent the manufacturing data. The output features (feature A and feature B) of the convolutional layers through Equations (4–7) are presented in the frequency domain (first and third columns) and the polar coordinate (second column). (a) Single-sinusoid-function case$.\ {\rm{The\ c}}$lass A signal is with fixed coefficients without noise ($u$ = 0), where $F_1^A = \ 300\,{\rm Hz}$, $\phi _1^A = \ 0$ and $a_1^A = \ 1$. Meanwhile, the coefficients of the class B signal are changed sequentially from the left plot to the right plot; the remaining two coefficients remain the same as the class A signal, where in the first plot $F_1^B = \ 600\,{\rm Hz}$, in the second plot $\ \phi _1^B = \frac{\pi }{2}\ $ and in the third plot $\ a_1^B = \ 5$. (b) The same coefficient settings as in (a), plus an additive Gaussian noise $u$ where $u\sim N( {0,0.05} )$ for all class A and B signals. (c) Sum of two sinusoid functions case. Class A signal is with fixed coefficients without noise ($u$ = 0), where $F_1^A = \ 400\,{\rm Hz}$, $F_2^A = \ 600\,{\rm Hz}$, $\phi _1^A = \ 0$, $\phi _2^A = \ 0$, $a_1^A = \ 1$ and $a_2^A = \ 1$. Similar to (a), the coefficients of the class B signal are changed sequentially from the left plot to the right plot, where in the first plot $F_1^B = \ 300\,{\rm Hz}$ and $F_2^B = \ 500\,{\rm Hz}$, in the second plot $\ \phi _1^B = \frac{\pi }{2}\ $ and $\ \phi _2^B = \frac{\pi }{2}\ $, and in the third plot $\ a_1^B = \ 5$, $\ a_2^B = \ 5$.

The time-series signal, as the most common form in manufacturing data, is constructed as the sum of harmonically related sinusoids and expressed by a Fourier series form (Equation (13)), based on [ISO 2041:2018] [41], with varying frequencies ${F_n}$ (associated with the fault frequencies, noise frequencies and resonance frequencies of mechanical components), phases ${\phi _n} \in [0,{\rm{\ }}2{\rm{\pi }}$], amplitudes ${a_n}$ (associated with damage levels on mechanical components) and/or white noise *u* (associated with environmental noises). Binary-classification experiments (Equation (13)), with class A signals ($F_n^A,\ \phi _n^A,\ a_n^B,\ u$) and class B signals ($F_n^B,\ \phi _n^B,\ a_n^B,\ u$), used to visualize the contribution of the convolutional layers, are conducted. Several effects with respect to different frequencies, phases, amplitudes and noises are shown in Fig. 4.

Starting from the basic single-sinusoid function (class A signal), the fault signals (class B signals) have varying features corresponding to different frequencies, phases or amplitudes from the left to the right plot, respectively (Fig. 4a). The frequency domain results in the first plot reveal that the extracted features (feature A and feature B) from convolutional layers are with the same frequencies as the input signals (class A and class B). This is a clue to classify manufacturing data with different frequency components due to wearing, breakage or deformation. The polar-coordinate result in the second plot shows an interesting phenomenon that the phase difference between the two features extracted is around $\frac{{\rm{\pi }}}{2}$, which is equal to the initial phase difference. The third plot shows the CNN ability to distinguish the amplitude difference of manufacturing data. Fault signals (class B signals) have five times the amplitude of the normal signal (class A signal); results demonstrate that the magnitudes of features after convolutional layers for normal and fault signals present the same proportional relationship as the initial input signals. In Fig. 4b, with an additive Gaussian noise compared to Fig. 4a, the results revealed that the CNN can ignore the redundant noise and extract the valuable information (almost the same features as Fig. 4a) for classification. Figure 4c shows a more complex case that is a combination of two sinusoid signals; features of fault signals after the convolutional operations (class B signals) can be also easily distinguished in both the frequency domain and the polar coordinate.

The final fault-prediction decision obtained by the CNN is a collective impact of all the coefficients discussed above with respect to signal frequencies, phases, amplitudes and biases. In this study, we attempt to give a plausible interpretation of the CNN framework for manufacturing data, from simple to complex cases. We reveal that CNN is successful for capturing the features of manufacturing problems due to the fact that the time-series signals (the most common form of manufacturing data) are compositional hierarchies.

### Robustness of the proposed CNN framework

To test the robustness of the proposed framework, different levels of noise are added to samples for test. For CWRU data set, additive noise, whose power varies from 0% to 500% of the original signal, is added; the prediction results for three classification tasks are shown in Fig. 1d. In addition, the intensities of the additive noise in other diagnosis applications are listed in Table 2. The classification applications still obtain high accuracies when the power of the additive noises is less than a certain level, which demonstrates the robustness of the proposed CNN framework. Detailed operations and the corresponding results of other cases can be found in the Supplementary Data.

**Table 2.** The range of added noise in each manufacturing application.

Data	Noise range (%)
Rolling bearing fault classification	[0–500]
Hydraulic system fault classification	[0–1]
Tool broken classification	[0–600]
Rolling bearing fault classification (own experiment)	[0–200]
Airplane girder fault classification	[0–20]
Aero engine blades processing classification	[0–150]
Gearbox fault classification	[0–500]

## DISCUSSION

In summary, we have demonstrated the effectiveness of the proposed framework for usage in manufacturing systems. Using a unified framework, we have tested the proposed deep-learning algorithm against a large number of critical diagnostic tasks in a variety of applications. The proposed end-to-end framework achieves the satisfactory accuracies reported for both the benchmark data sets and our own data sets. The interpretation ability of the fault prediction in the CNN model provides valuable information for understanding why deep learning can make a diagnosis decision on manufacturing data with different frequencies, amplitude and phases. With the designed hardware implementation specified in the Supplementary Data, our proposed algorithm framework could be easily applied to data sets in other industry applications.

This framework entails some limitations, which shall be listed as our future work. The limitations include, first of all, the method has a few hyper-parameters to tune, in order to achieve the best performance; a cross-validation or optimization algorithm could be used to select the hyper-parameters, such as the kernel size, the number of strides and the number of layers. Second, given the number of parameters in the constructed model, it requires a large amount of data to train; this may not be feasible for certain applications.

## METHODS

### Data sets

The data sets used in this manuscript are of the following types: open-accessible data, competition data, experimental data collected in our lab and real production data provided by industrial partners with permission. These data sets are composed of sensory-current signals, force signals, vibration signals or acoustic-emission signals, or their combinations, which are processed for the classification or regression tasks.

### Main idea

We convert practical problems into supervised classification and regression tasks, and solve them using a deep-learning technique. An end-to-end algorithm is proposed to automatically discover the hidden features needed for learning and prediction without prior knowledge. We develop a novel framework based on CNN that performs fault diagnosis and prediction and regression based on the raw data. We constructed a fully automated closed-loop system: a CNN model is fed with the sensory measurements and automatically extracts the features for classification or prediction. The results learned by the CNN are then fed back to the machine for decision-making, such as whether a maintenance action is required.

### Pre-processing

We normalize the measurements in each data set in several ways as detailed in the Supplementary Data. More specifically, for data sets with a small number of time-course measurements, such as CWRU-bearing data set, we divided the total features to a constant length in each sample without affecting the periodicity of the data. For prediction tasks, such as Case 8, the data set is transformed by a standardization as specified in the Supplementary Data.

### Parameter-tuning

We propose to fine-tune the CNN model according to different classification and prediction objectives, with a fixed Max-pooling size of $1 \times 2$. To extract fewer features, stride sizes (i.e. the sliding-window size) in CNN models are set to be, for example, 500 or 1000 in a data sequence with tens of thousands of dimensions, 100 or 200 in a data sequence with thousands of dimensions and adjusted according to specific applications. The basic components of the proposed CNN model are stacked with input data, CNN layers and a fully connected layer (including an output layer). For classification problems, the number of nodes *N* in the output layer is equal to the number of fault types. For regression problems, *N* is set to 1. For detailed model parameters of difference applications, refer to the Supplementary Data.

### Convolutional neural networks

In the proposed framework, CNNs consist of convolutional layers, Max-pooling layers, a flatten layer and fully connected layers with a final N-way prediction layer. In essence, the CNN uses raw data $ I \in {\mathbb{R}^{\rm{k}}}$ as inputs and outputs are classification or regression results $\hat{y}$, i.e.

(4) \begin{eqnarray*} \hat{y} &=& {\rm act( FCN( {\rm Flatt}({pool}} \nonumber\\ &&{\rm ({ReLU}({conv}(I))))))}. \end{eqnarray*}

The convolutional layer (conv) uses a number of filters to discretely convolve with the input data. We define a weight vector $H \in {\mathbb{R}^{\rm{m}}}$, a data vector $I \in {\mathbb{R}^{\rm{k}}}{\rm{\ }}$computed from raw data and a constant $b$ of a bias. In a convolutional process, stride is the distance between two sub-convolution windows and we define it as parameter *d*. We define the *i*_*th*_ sub-vector of $I$, i.e.${\rm{\ \ }}{I^{( i )}} = {[ {{I^{1 + ( {i - 1} )d}}, {I^{2 + ( {i - 1} )d}},\ \ldots ,\ {I^{m + ( {i - 1} )d}}} ]^T}$ ($i=1, 2, \ldots, \frac{{k - m}}{d} + 1)$. The idea of a 1D convolution is to take the product between the vector $H$ and the sub-vector ${I^{( i )}}$ of raw data, which reads as follows:

(5) \begin{equation*}{S^{\left( {\rm{i}} \right)}} = {I^{\left( {\rm{i}} \right)}}\,{\rm{\ *}}\,H + b\ = \mathop \sum \limits_{j = 1}^m {I^{j + \left( {i - 1} \right)d}}{H^j} + b,\end{equation*}

where ${H^j}$ is the *j_th_* element of vector *H*, *j = *1,2,$\ldots$, *m*. When conducting a convolutional process, the number of filters (different filters have different initial vector *H*) are set to determine the depth of the convolutional results. Since the process of convolution between each filter and data uses weight sharing, the number of training parameters and complexity of the model are greatly reduced. As a result, computational efficiency is improved.

An activation function named the Rectified Linear Unit (ReLU) is followed by each convolutional layer, which has the following form:

(6) \begin{equation*} {U^i} = {\rm ReLU( {{S^{( i )}}})} \buildrel \Delta \over = {\rm max} ({0,{S^{( i )}}}). \end{equation*}

ReLU avoids gradient vanishing with respect to other functions when the optimizer calculates the gradient descent, while guaranteeing the sparsity in convolutional networks, which significantly reduces the training time compared with other activation functions. The above operations lead to the results of $U = {[ {{U^1}, \ldots ,{U^i},\ldots, {U^{\frac{{k - m}}{d} + 1}}} ]^T}$.

Then Max pooling (pool) is chosen here:

(7) \begin{eqnarray*} &&{\rm pool}({U^i}): = \max _{\ell = 1}^p\,\,{U^{\ell + (i - 1)e}},\\ &&\forall i =1,2, \ldots ,\frac{{k - m}}{d} + 1,\end{eqnarray*}

where *p* is the pooling size and *e* is the stride size.

After convolution and pooling, the data are fed into a flattened layer (Flatt); data are transformed into a 1D structure in Flatt, denoted as $F\ = [ {{F_1},\ {F_2},\ \ldots,\ {F_q}} ];\ q$ is the length of the data after the flattened layer to facilitate data processing in the fully connected layers (FCNs). Then, the FCNs combined with the ReLU activation function is utilized to realize the dimensionality reduction, which can be written as:

(8) \begin{equation*} O = {\rm{\ ReLU}} ({W} \cdot {F}), \end{equation*}

where *W* are the weights of the FCNs, $O\ = \ [ {{O_1},\ {O_2},\ \ldots ,\ {O_N}} ]$ is the output of the FCNs and ‘$ \cdot $’ is the dot product. *N* is the number of faulty types in the classification task and *N* = 1 in the regression task.

The output-activation function (act) uses a softmax function for the classification problem or a sigmoid function for the regression problem. For classification, the estimated result $\hat{y} = \ {\rm act}( O )$ can be shown as:

(9) \begin{equation*} {\hat{y}_n} = \frac{{{e^{{O_n}}}}}{{\Sigma _{j = 1}^N{e^{{O_j}}}}}\ ,{\rm{\ \ for}}\ \ n\ = {\rm{\ }}1,2, \ldots N. \end{equation*}

And, for regression,$\ \ \hat{y} = \ {\rm act}( O )$ is:

(10) \begin{equation*} \hat{y} = \frac{1}{{1 + {e^{ - O}}}}\ . \end{equation*}

In the training process, to minimize the difference between the predicted scores and the ground labels in the training data, cross-entropy ${L_{ce}}{\rm{\ }}$and least-squares ${L_{ls}}$ are chosen as the loss function for the classification problem and the regression problem, respectively, which are defined in Equations (11) and (12):

(11) \begin{eqnarray*} &&{\rm{\ }}{L_{ce}} &=& {\rm{\ }} -\!\frac{1}{q}\mathop\sum \limits_{i =1}^q\mathop\sum \limits_{n=1}^N1\{ {\ {y^{( i )}} = {\rm{\ }}n} \}\log {\hat{y}^{( i )}}\nonumber\\ && +\,\, ( {1 - 1\{ {\ {y^{( i )}} = {\rm{\ }}n} \}} )\log ( {1 - {{\hat{y}}^{(i)}}} ),\nonumber\\ \end{eqnarray*}

(12) \begin{equation*} {\rm{\ }}{L_{ls}} = \frac{1}{q}\Sigma _{i = 1}^q{\left( {{y^{\left( i \right)}} - {{\hat{y}}^{\left( i \right)}}} \right)^2}, \end{equation*}

where ${y^{( i )}}$ is the real output of the *i*_*th*_ training measurement and $q$ the total number of training measurements. The term $1\{ {\ {y^{( i )}} = \ n} \}$ in Equation (11) is the logical expression that always returns either 0 or 1.

Once the loss function has been chosen, we use standard optimizers such as Stochastic Gradient Descent (SGD) [42] or Adam [43] for parameter training in back-propagation to update the weights. The final CNN model weights refresh until the predefined maximum iteration to yield a lower loss.

### Interpretation of the CNN model for manufacturing data

In order to interpret how the CNN model learns from manufacturing data, we consider a time-series signal with the most common form of manufacturing data, which is modeled as the sum of harmonically related sinusoidal functions:

(13) \begin{eqnarray*} {v^{\left( i \right)}}& =& \sum\nolimits_{n = 1}^N {a_n}\sin \left({2\pi {F_n}{x}^{(i)}} + {\phi_n}\right) \nonumber\\ && + {u^{\left( i \right)}}\ ,{\rm{\ for\ }}{x^{\left( i \right)}} \in \left[ {0,{\rm{\ }}0.4} \right]{\rm{\ and\ }} \nonumber\\ i & =& {\rm{\ }}0,{\rm{\ }}1, \ldots ,{\rm{\ }}4095, \end{eqnarray*}

where ${v^{( i )}}$ is the magnitude of the *i*_*th*_ measurements and $u$ is the Gaussian noise. The magnitudes of the time-series signal ${v^{( i )}}$ corresponds to four coefficients: the sinusoid frequencies ${F_n}$, the amplitudes ${a_n}$, the phases ${\phi _n} \in [ {0,{\rm{\ }}2{\rm{\pi }}} ]$ and the Gaussian noise $u$, respectively.

To provide a clear interpretation of convolutional layers, we conducted binary-classification (class A and class B) experiments by changing one of the four coefficients, while keeping the other three coefficients unchanged (Fig. 4). For each binary-classification task, we duplicated 100 times the class A signal ${v_A} = {[ {v_A^{( 0 )},\ \ldots ,\ v_A^{( i )},\ \ldots ,\ v_A^{( {4095} )}} ]^T}\ $ and class B signal ${v_B} = {[ {v_B^{( 0 )},\ \ldots ,\ v_B^{( i )},\ \ldots ,\ v_B^{( {4095} )}} ]^T}\ $ as the samples for training and test. Randomly, 90% of samples are used for model training and the other 10% for samples test; 100% accuracy is obtained. Class A and B signals are processed to the convolutional operation using Equations (4–7) to obtain the features for visualization. Figure 4 analyses the extracted features in the frequency domain or the polar coordinate corresponding to different coefficients in Equation (13).

### Cross-validation

The random-subsets approach is used for dividing the training set and the test set in all the classification tasks. For the data sets that have small sample sizes, such as tool-broken data, blades-processing data and gearbox data, we randomly split the data set into 80% (training) and 20% (test). For other classification tasks with large sample sizes, we randomly split the data set into 90% for training and 10% for test. For the CWRU data (Case 1), bearing data (Case 4) and gearbox data (Case 7), two other cross-validation methods (i.e. contiguous block and independent sequence) are used to verify the effectiveness of the framework. The contiguous-block method utilizes the latter part of samples, which are reconstructed from one long time series as the test set and the other part as the training set. For the contiguous-block method, a 10%, 20%, 30%, 40% and 50% test scheme is used. The first (100–X)% of part of the time series is used for training and the rest (X% of the data) is used for the test. The independent-sequence method divides the training set and the test set as independent time series. For three prediction tasks, leave-one-out cross-validation is used. For instance, NASA battery data have three degradation batteries and we randomly use two for training and the other one for test each time, then three models in total are trained to validate the three-battery data.

### Robustness analysis

In order to verify the robustness of the proposed method in each classification task, additive white Gaussian noise with power *P*, which is proportional to the power of the original sample ${P_0}\ $with a coefficient *S*, is added to each sample. The noise power *P* can be derived from the following expression:

(14) \begin{equation*} P\ = {\rm{\ }}S{\rm{\% }} \cdot {\rm{\ }}{P_0} = {\rm{\ }}S{\rm{\% }} \cdot \frac{1}{k} \cdot \sum\limits_{j\ = {\rm{\ }}1}^k {{{({I^j})}^2}}, \end{equation*}

where *I* is the raw data sample and *k* is the length of the raw data. The random-subsets method of cross-validation is used in each classification application and the according accuracy variance with the power of the noise is given in each application.

### Data-availability statement

The bearing-fault and aircraft-girder data sets can be downloaded at the Manufacturing Network Platform that we built: http://mad-net.org:8765/. The CRWU-bearing data set is available at http://www.eecs.cwru.edu/laboratory/bearing. The NASA tool- wear data set can be downloaded from https://ti.arc.nasa.gov/tech/dash/groups/pcoe/prognostic-data-repository/. NASA and CALCE battery data sets are available at http://ti.arc.nasa.gov/project/prognostic-data-repository and https://web.calce.umd.edu/batteries/data.htm#, respectively. The hydraulic-system data set is available at https://archive.ics.uci.edu/ml/datasets/Condition+monitoring+of+hydraulic+systems#. The experimental data including gearbox, aero-engine-blade-processing data sets and tool-broken data sets are available from the corresponding author upon request.

## Supplementary Material

nwz190_Supplemental_File

## References

[1] Manyika J , Sinclair J, Dobbs R et al. Manufacturing the future: the next era of global growth and innovation. New York: McKinsey Global Institute, 2012, 1–184.

[2] Cui B , Mei H, Ooi BC. Big data: the driver for innovation in databases. Natl Sci Rev 2014; 1: 27–30.

[3] Kusiak A . Smart manufacturing must embrace big data. Nature 2017; 544: 23–5. 28383012 10.1038/544023a

[4] Liu R , Yang B, Zio E et al. Artificial intelligence for fault diagnosis of rotating machinery: a review. Mech Syst Signal Process 2018; 108: 33–47.

[5] Gao Z , Cecati C, Ding SX. A survey of fault diagnosis and fault-tolerant techniques-part I: fault diagnosis with model-based and signal-based approaches. IEEE Trans Ind Electron 2015; 62: 3757–67.

[6] Mei H , Hao D, Zhang L et al. A static approach to prioritizing JUnit test cases. IEEE Trans Softw Eng 2012; 38: 1258–75.

[7] Jia F , Lei Y, Lin J et al. Deep neural networks: a promising tool for fault characteristic mining and intelligent diagnosis of rotating machinery with massive data. Mech Syst Signal Process. 2016; 72–73: 303–15.

[8] Yin S , Ding SX, Xie X et al. A review on basic data-driven approaches for industrial process monitoring. IEEE Trans Ind Electron 2014; 61: 6414–28.

[9] Yuan Y , Tang X, Zhou W et al. Data driven discovery of cyber physical systems. Nat Commun 2019; 10: 4894. 31653832 10.1038/s41467-019-12490-1 PMC6814766

[10] Yuan Y , Zhang HT, Wu Y et al. Bayesian learning-based model-predictive vibration control for thin-walled workpiece machining processes. IEEE/ASME Trans Mechatronics 2017; 22: 509–20.

[11] Cai J , Luo J, Wang S et al. Feature selection in machine learning: a new perspective. Neurocomputing 2018; 300: 70–9.

[12] Rauber TW , De F, Varejão FM. Heterogeneous feature models and feature selection applied to bearing fault diagnosis. IEEE Trans Ind Electron 2015; 62: 637–46.

[13] Liu H , Liu C, Huang Y. Adaptive feature extraction using sparse coding for machinery fault diagnosis. Mech Syst Signal Process 2011; 25: 558–74.

[14] Shao H , Jiang H, Zhang H et al. Electric locomotive bearing fault diagnosis using a novel convolutional deep belief network. IEEE Trans Ind Electron 2018; 65: 2727–36.

[15] Ding X , He Q. Energy-fluctuated multiscale feature learning with deep ConvNet for intelligent spindle bearing fault diagnosis. IEEE Trans Instrum Meas 2017; 66: 1926–35.

[16] Krizhevsky A , Sutskever I, Hinton GE. ImageNet classification with deep convolutional neural networks. Adv Neural Inf Process Syst 2012; 60: 1097–105.

[17] Esteva A , Kuprel B, Novoa RA et al. Dermatologist-level classification of skin cancer with deep neural networks. Nature 2017; 542: 115–8. 28117445 10.1038/nature21056 PMC8382232

[18] Li W , Field KG, Morgan D. Automated defect analysis in electron microscopic images. npj Comput Mater 2018; 4: 36.

[19] Abdel-Hamid O , Mohamed A, Jiang H et al. Convolutional neural networks for speech recognition. IEEE/ACM Trans Audio Speech Lang Process 2014; 22: 1533–45.

[20] Lecun Y , Bengio Y, Hinton G. Deep learning. Nature 2015; 521: 436–44. 26017442 10.1038/nature14539

[21] Bearing Data Center . Case Western Reserve University Seeded Fault Test. http://csegroups.case.edu/bearingdatacenter/pages/download-data-file (28 August 2018, date last accessed).

[22] Helwig N , Pignanelli E, Schutze A. Condition monitoring of a complex hydraulic system using multivariate statistics. Conference Record—IEEE Instrumentation and Measurement Technology Conference 2015, 210–5.

[23] Mad Net . Mad Data Set. http://mad-net.org:8765/explore.html?t=0.597370213951085 (28 August 2018, date last accessed).

[24] He Q , Guo Y, Wang X et al. Gearbox fault diagnosis based on RB-SSD and MCKD (in Chinese). China Mech Eng 2017; 28: 1528–34.

[25] Agogino A , Goebel K. ‘Milling Data Set’, NASA Ames Prognostics Data Repository (https://ti.arc.nasa.gov/tech/dash/groups/pcoe/prognostic-data-repository/), NASA Ames Research Center, Moffett Field, CA, 2007.

[26] Saha B , Goebel K. ‘Battery Data Set’, NASA Ames Prognostics Data Repository (https://ti.arc.nasa.gov/tech/dash/groups/pcoe/prognostic-data-repository/), NASA Ames Research Center, Moffett Field, CA, 2007.

[27] He W , Williard N, Osterman M et al. Prognostics of lithium-ion batteries based on Dempster-Shafer theory and the Bayesian Monte Carlo method. J Power Sources 2011; 196: 10314–21.

[28] El-Thalji I , Jantunen E. A summary of fault modelling and predictive health monitoring of rolling element bearings. Mech Syst Sig Process 2015; 60: 252–72.

[29] Bearing Data Center . Apparatus & Procedures. https://csegroups.case.edu/bearingdatacenter/pages/apparatus-procedures (28 August 2018, date last accessed).

[30] Eigenvector Documentation Wiki . Using Cross-Validation. http://wiki.eigenvector.com/index.php?title=Using_Cross-Validation (28 August 2018, date last accessed).

[31] Van Der L , Hinton G. Visualizing data using t-SNE. J Mach Learn Res 2008; 9: 2579–605.

[32] Sebastiani F . Machine learning in automated text categorization. ACM Comput Surv 2002; 34: 1–47.

[33] Boutell MR , Luo J, Shen X et al. Learning multi-label scene classification. Pattern Recognit 2004; 37: 1757–71.

[34] Tsoumakas G , Katakis I. Multi-label classification. Int J Data Warehous Min 2007; 3: 1–13.

[35] Wang F , Stelson KA. An efficient fan drive system based on a novel hydraulic transmission. IEEE/ASME Trans Mechatronics 2015; 20: 2234–41.

[36] Deppen TO , Alleyne AG, Stelson K et al. An energy management strategy for a hydraulic hybrid vehicle. In: *2012 American Control Conference (ACC)*. Montreal: IEEE, 2012, 1335–41.

[37] Ma G , Zhang Y, Cheng C et al. Remaining useful life prediction of lithium-ion batteries based on false nearest neighbors and a hybrid neural network. Appl Energy 2019; 253: 113626.

[38] Dong G , Chen Z, Wei J et al. Battery health prognosis using brownian motion modeling and particle filtering. IEEE Trans Ind Electron 2018; 65: 8646–55.

[39] Sturm I , Lapuschkin S, Samek W et al. Interpretable deep neural networks for single-trial EEG classification. J Neurosci Methods 2016; 274: 141–5. 27746229 10.1016/j.jneumeth.2016.10.008

[40] Nielsen AAK , Voigt CA. Deep learning to predict the lab-of-origin of engineered DNA. Nat Commun 2018; 9: 3135. 30087331 10.1038/s41467-018-05378-z PMC6081423

[41] ISO 2041:2018(en) . Mechanical vibration, shock and condition monitoring—Vocabulary. https://www.iso.org/obp/ui/#iso:std:iso:2041:ed-4:v1:en (28 August 2018, date last accessed).

[42] Bottou L . Stochastic gradient descent tricks. Lect Notes Comput Sci 2012; 7700: 421–36.

[43] Kingma DP , Ba J. Adam: a method for stochastic optimization. In: International Conference on Learning Representations. San Diego: Elsevier, 2015.

