# Supplementary material for: A general end-to-end diagnosis framework for manufacturing systems
Source: Natl Sci Rev. 2019 Nov 21;7(2):418–29. doi: 10.1093/nsr/nwz190 (PMC8289032; doi:10.1093/nsr/nwz190)
Supplement: nwz190_Supplemental_File [file nwz190_supplemental_file.docx]

**Supplementary Information**

# Case 1: Anomaly detection of rolling element bearings (REBs) using classification

*Problem formulation:* To improve production quality, shorten repairing time, and avoid human casualties, modern manufacturing industry shifts its maintenance strategies from time-based maintenance to condition-based maintenance. Bearing failure is one of the foremost causes of breakdown in rotating machinery [1]. Therefore, it is of great importance to detect or even predict bearing faults, which could avoid the breakdown of rotating machinery. Vibration signal, used in this case, is the most reliable and effective source for bearing fault diagnosis [2]. Faulty bearing vibration signals are usually caused by localized defects in three components, namely rolling elements, the outer race, and the inner race of the bearing. Rolling elements transform sliding friction to rolling friction in a working rotator. The bearing outer race, including a groove for rolling elements to rotate on right tracks, is fixed on the machine shell to support the working rolling elements. The inner race, which is fixed on the spindle, drives rolling elements rotating along the groove. When it gets close to the end of bearings’ lifetime, deformation, crack, and burning between these three components may cause spindle deviation and further exert serious damage to the mechanical system. Therefore, it is key to investigate bearing faults classification problem on these bearing components, i.e., localize the fault from vibration signals.

*Introduction and properties of data set:* The bearing data set provided by Case Western Reserve University (CWRU) data center [3] has been considered as a benchmark for bearing faults diagnosis problem. It is employed to validate the effectiveness of the proposed method. An experiment platform (see in Fig. S2 (d)) is used to collect the vibration signals for the detection of defects on three bearings with different fault diameters (7-mil, 14-mil, and 21-mil (1 mil=0.001 inches)). Properties of this data set include the following:

- We choose the vibration data acquired by accelerometers for fault diagnosis.
- It is ideal for fault diagnosis problem due to a sufficient number of faulty types, which is composed of three fault diameters of bearings corresponding to three different components of the bearing. More precisely, faulty vibration signals are acquired by accelerometers from the inner race, outer race, and rolling elements for each fault diameter of the bearing.
- Three models are trained for this fault diagnosis problem, including a binary classification model (normal and faulty), a four-way classification model (normal and main faults for three components respectively), and a ten-way classification model (normal and main faults for three components with different bearing fault diameters).
- The frequency resolution of this data set (12 kHz) is sufficiently high to obtain a large quantity of time-course measurements for classification. The data set originally consists of 4 normal samples and 52 faulty samples. Three of the normal samples have 480,000 measurements and the other one has 240,000 measurements, while every faulty sample has 120,000 measurements. The oscillation of the normal vibration signal and the other nine types of faulty vibration signals in this data set (first 6000 points of each signal) are presented in Fig. S1.


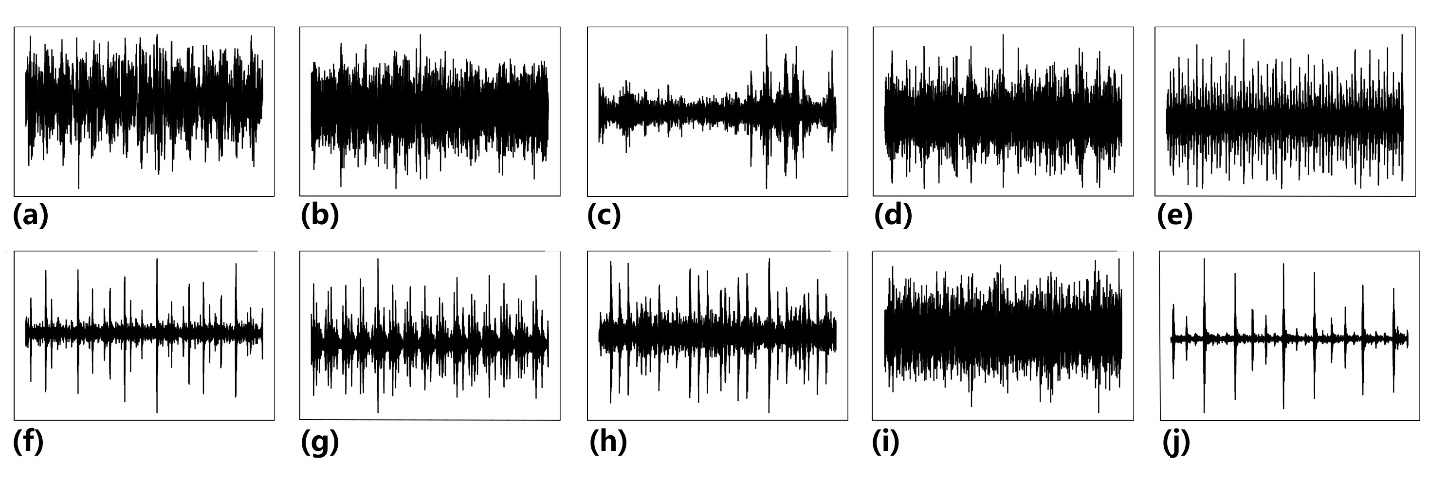


Fig. S1. The oscillation of the normal vibration signal and the other nine types of fault vibration signals (first 6000 points of each signal): (a) normal, (b) rolling elements with 7-mil fault diameters, (c) rolling elements with 14-mil fault diameters, (d) rolling elements with 21-mil fault diameters, (e) inner race with 7-mil fault diameters, (f) inner race with 14-mil fault diameters, (g) inner race with 21-mil fault diameters, (h) outer race with 7-mil fault diameters, (i) outer race with 14-mil fault diameters, (j) outer race with 21-mil fault diameters.

*Data pre-processing:* Assuming the faulty signal is a stationary process [4], we here reshape the samples to make each sample have 6000 measurements consistently. In total, 1320 samples are constructed from the original data set. Details of the original data and the pre-processed data are summarized in Table S1. To evaluate model performance, the obtained entire pre-processed data set is then randomly split into two parts: 90% for training (1188 samples), and the rest 10% for test (132 samples).

Table S1: Original data and pre-processed data in CWRU data set.

|  | Original data | | Pre-processed data | |
| --- | --- | --- | --- | --- |
|  | Measurement | Sample Number | Measurement | Sample Number |
| Normal Signal | 480,000 | 3 | 6000 | 240 |
|  | 240,000 | 1 | 6000 | 40 |
| Faulty signal | 120,000 | 52 | 6000 | 1040 |

*Prediction results:* We fit the training data using a CNN model and test the model effectiveness on the test data set. Fig. S2(a)-(c) show the classification results for the faulty bearing vibration data. Across the three tests, it is observed that the models all achieve 100% (132 of 132 test samples) faults classification. A confusion matrix is a common tool to evaluate the performance of classification results [5]. For a binary classification problem, the confusion matrix could be characterized by the numbers of true positives (TP), false positives (FP), false negatives (FN), and true negatives (TN), where TP represents the positive samples are correctly predicted, FP means the positive samples are incorrectly predicted, FN is that the negative samples are incorrectly predicted, and TN means the negative samples are correctly predicted. For a multi-classification problem, the number in each grid represents the number of the samples are classified into other classes. Fig. S3 contains the confusion matrices of the classification results to demonstrate the excellent performance of the proposed classifier. Detailed tuning parameters, such as kernel size and max pooling size, are presented in Fig. S4.


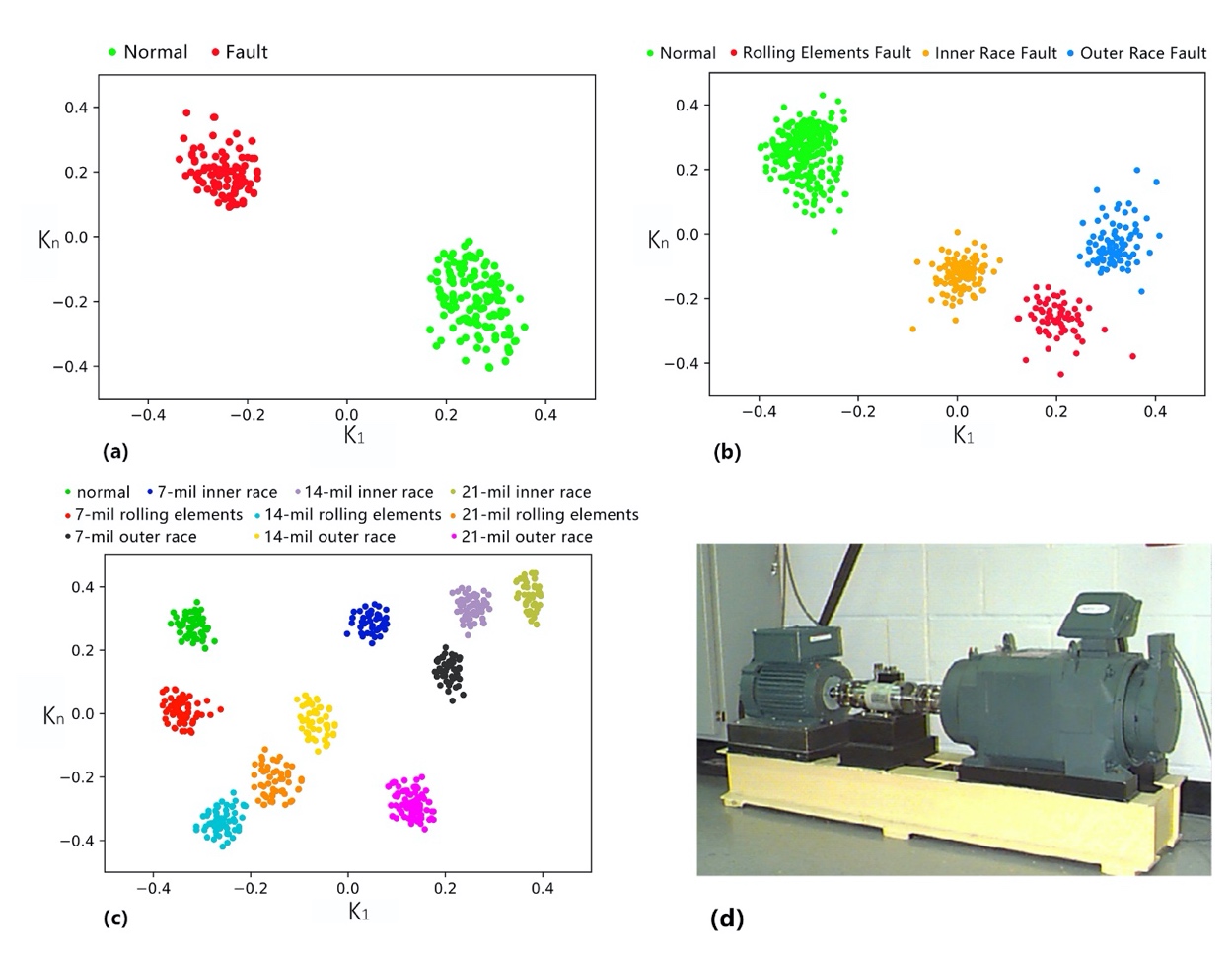


Fig. S2. Sketch maps of classification results on test data sets of CWRU bearing are shown in two-dimensional coordinate system (K_1_ and K_n_ are two virtual features). (a) Binary classification task in terms of normal and fault. (b) Four-way classification task in terms of normal, rolling elements fault, inner race fault, and outer race fault. (c) Ten-way classification task in terms of normal and other nine types of faults on three bearing with difference sizes. (d) The experiment platform in CWRU bearing data center.


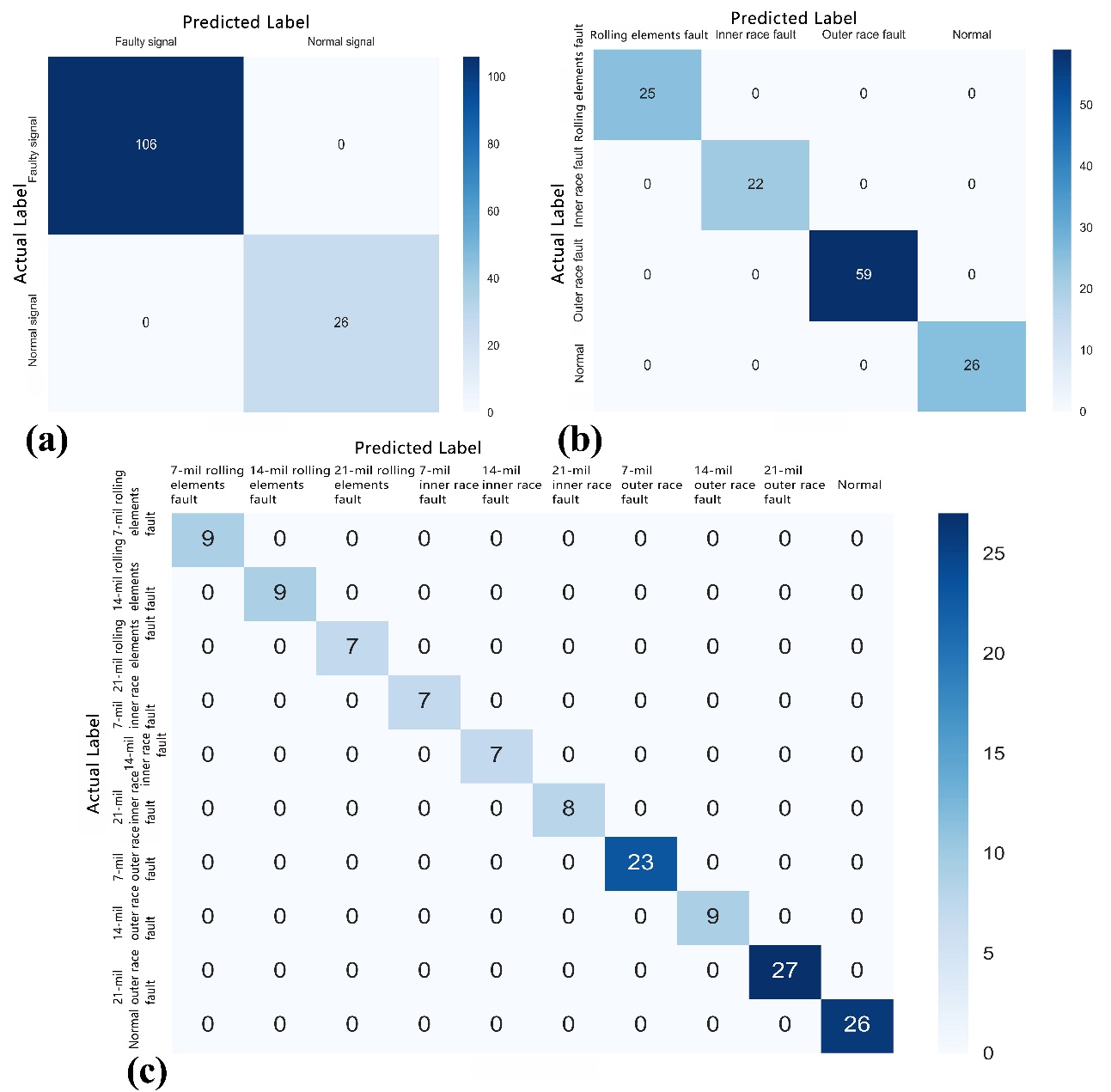


Fig. S3. Confusion matrices of CWRU bearing fault classification: (a) binary classification, (b) four-way classification, and (c) ten-way classification.

*Feature verification:* To validate the effectiveness of learned features of the CNN model, 3712 intermediate features of a random fully connected layer after 100 iterations are gathered. A linear classifier SVM is then followed for evaluating the effectiveness of these features. However, SVM classification on 3721 features incurs a high computational cost. With the aim of increasing the computational efficiency, the Fisher Score is introduced for feature reduction and the algorithm is outlined in Table S2. Fisher Score selects the features by calculating the score value of every feature such that the feature values of samples within the same class are small while the feature values of samples from different classes are large [6]. Through pruning scores under a certain threshold, we reserve 30%, 15%, and 5% features from 3712 dimensions before using linear classifier SVM, and ultimately get the accuracy of 100%, 100%, and 87%. This experiment shows that, before the output layer, the main fault features of the bearings have already been successfully extracted.

Table S2: The steps of Fisher Score to reduce dimension in the feature space.


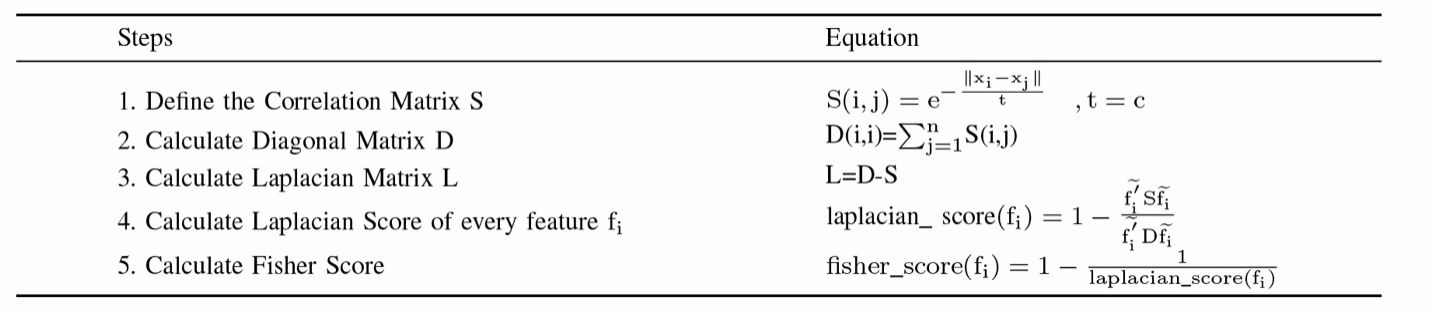


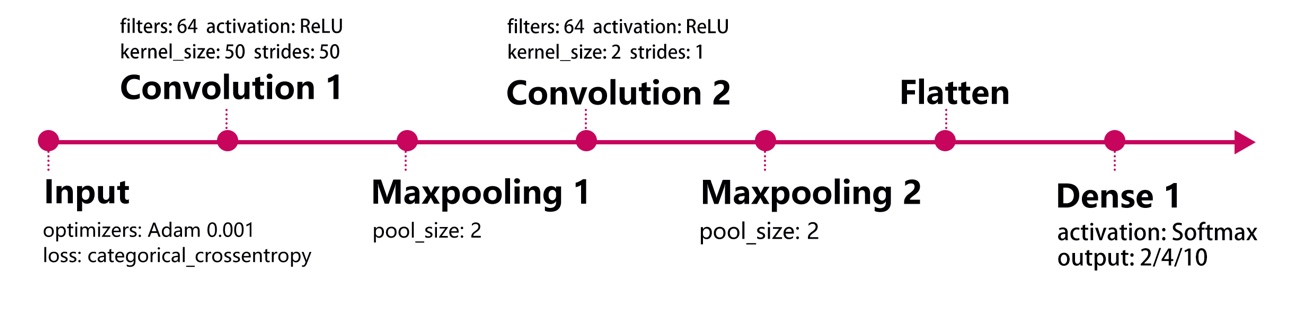


Fig. S4. CNN structure and parameters for the CWRU bearing classification task.

*Comparison and discussion:* To illustrate the performance of our proposed framework, other state-of-the-art methods used for the same data set are employed for comparison. The results are given in Table S3. Our proposed CNN model achieves 100% accuracies, which outperforms the conventional methods (e.g., Wavelet and Permutation Entropy methods). Meanwhile, compared with ANN, DBN or SVM methods only applied to one or two kinds of classification task, the proposed CNN method can handle three diagnosis tasks within a unified framework, demonstrating its wide applicability.

Table S3: The comparative results on rolling bearing data set.

| Authors | Methods | Classified states | Accuracy |
| --- | --- | --- | --- |
| **Binary task** | **Ours** | **2** | **100%** |
| **Two-way task** | **Ours** | **4** | **100%** |
| **Ten-way task** | **Ours** | **10** | **100%** |
| Shao *et al.* [7] | DBN | 2 | 100% |
| Shao *et al.* [7] | DBN | 10 | 90% |
| Samanta *et al.* [8] | ANN, SVM, GA | 2 | 92% |
| Yang *et al*. [9] | ANN | 4 | 100% |
| Sreejith *et al.* [10] | ANN | 4 | 100% |
| Chebil *et al*. [11] | Wavelet | 10 | 95% |
| Vakharia *et al.* [12] | Permutation Entropy | 4 | 97.5% |

*Robustness analysis:* To verify the robustness of the proposed approach, additive Gaussian noise, whose power *P* varies from 0% to 500% of each original signal power $P_{0}$ is added (see in Fig. S5 (a-c)). Similarly, the obtained entire pre-processed data set is then randomly split into two parts: 90% for training (1188 samples) and the rest 10% for test (132 samples). As the power of the noise increases, the accuracies of the three classification models change (see in Fig. S5 (d)). The accuracies all surpass 98% when the noise ratios are smaller than or equal to 100%.


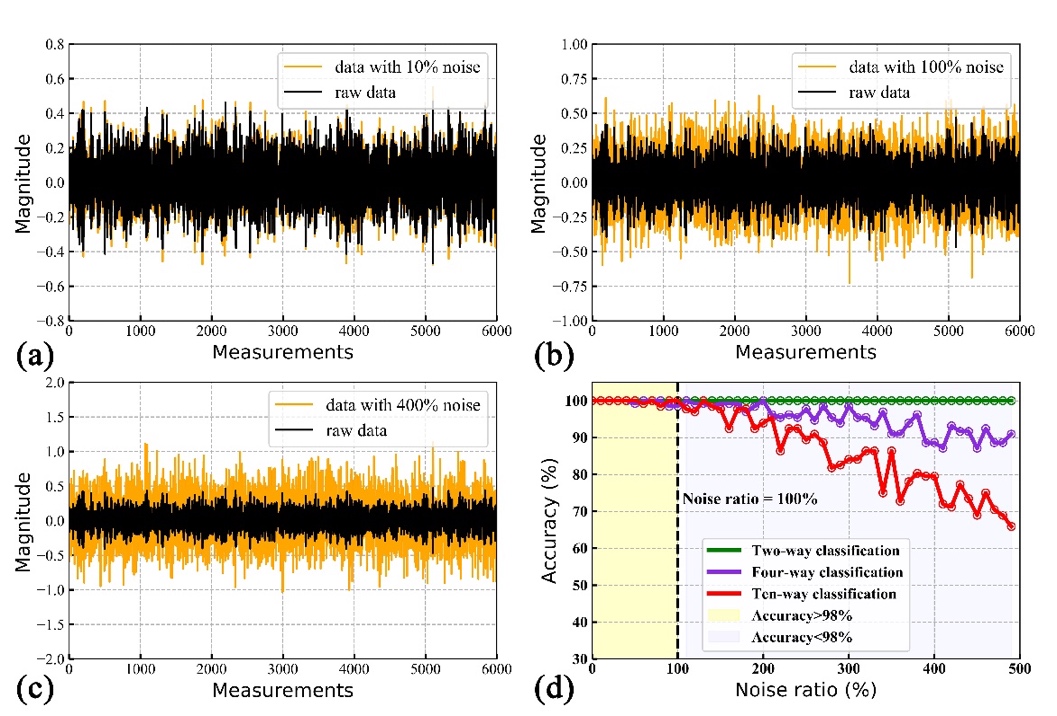


Fig. S5. Comparisons between raw signal and (a) raw signal plus additive Gaussian noise whose P=10% $\cdot P_{0}$; (b) raw signal plus additive Gaussian noise whose P=100% $\cdot P_{0}$; (c) raw signal plus additive Gaussian noise whose P=400% $\cdot P_{0}$. (d) Curves of accuracies with noise ratio for three classification models.

*Cross validation:* Except the random subsets method, we also employ contiguous block and independent sequence methods of cross-validation to eliminate the time dependency of training samples and test samples, hence to verify the effectiveness of the model. Contiguous block method utilizes the latter part of samples which are reconstructed from one long time-series as test set while the other part as training part; thus, the test set would not be mixed in training set. Independent sequence method partitions training set and test set from entire time-series. In this case, the training set and test set are obtained based on rotating speeds, where data with rotating speeds of 1797 rpm, 1750 rpm, and 1730 rpm are used for training and data with rotating speeds of 1772 rpm are used for test. We summarize the results of different cross-validation methods in Table S4.

Table S4: Three cross-validation methods (random subsets, contiguous block and independent sequence) were employed to verify the effectiveness of the model in the CWRU data.

|  | Random subsets | Independent sequence | Contiguous block | | | | |
| --- | --- | --- | --- | --- | --- | --- | --- |
|  |  |  | 10% test | 20% test | 30% test | 40% test | 50% test |
| Two-way  classification | 100% (132/132) | 100% (340/340) | 100% (132/132) | 100%  (262/264) | 100%  (396/396) | 100% (528/528) | 100% (660/660) |
| Four-way classification | 100% (132/132) | 100% (340/340) | 100% (132/132) | 99.24% (262/264) | 97.22% (385/396) | 95.83% (506/528) | 96.21% (635/660) |
| Ten-way classification | 100% (132/132) | 98.82% (336/340) | 99.24% (131/132) | 98.86% (261/264) | 98.48% (390/396) | 96.21% (508/528) | 96.67% (638/660) |

# Case 2: Hydraulic system fault classification

*Problem formulations:* In recent years, hydraulic system, which transfer force from one link to another using hydraulic fluid, have provided the power behind a large number of modern automation systems [13]. Their indispensability means that constant attention must be focused on condition monitoring in order to keep system running in safety. Thus, condition monitoring of hydraulic system has gained increasing importance in multiple areas such as electrical industries and yet the problem of how to do so has still not been solved effectively.

*Introduction and properties of data set:* The condition monitoring of hydraulic system data set [14] provided was obtained experimentally through a hydraulic test rig consisting of a primary working circuit and a secondary cooling-filtration circuit [15], which are connected via the oil tank. The system periodically repeats constantly load cycles of 60 seconds, and process values such as pressures, volume flows, and temperatures are measured. At the same time, the condition of four hydraulic components (cooler, valve, pump, and accumulator) is varied. Properties of this data set include the following:

- It contains different kinds of raw process sensor data (pressure sensors, motor power sensors, volume flow sensors, temperature sensors, vibration sensors, efficiency factor, virtual cooling efficiency sensors, and virtual cooling power sensors) from measurements taken at the same point in time.
- The sensors were read with different sampling rates, leading to different numbers of measurements per sensor, while we choose only PS1 (i.e., the first pressure sensor) data for condition classification.
- The data set contains five different condition classes (cooler condition, valve condition, internal pump leakage, hydraulic accumulator, and stable flag).
- The frequency resolution of PS1 pressure sensor is 100 Hz and we obtain input sequences with 6000 measurements of each sample. 2205 samples are acquired in total. Each sample contains five condition classes, while we only consider the first four conditions (except stable flag) for classification.
- Four models are trained for this condition classification problem, including a three-way classification for cooler condition (close to total failure, reduced efficiency, and full efficiency), a four-way classification for valve condition (optimal switching behavior, small lag, severe lag, and close to total failure), a three-way classification model for internal pump leakage (no leakage, weak leakage, and severe leakage), and a four-way classification model for hydraulic accumulator (optimal pressure, slightly reduced pressure, severely reduced pressure, and close to total failure).

*Prediction results:* We achieve excellent accuracies of 100% in both cooler condition and valve condition classifications. The pump leakage and hydraulic accumulator classifications also give satisfactory accuracies, at 98.19% and 99.55%, respectively. Fig. S6 shows the hyper-parameters in hydraulic system condition classification. Fig. S7 presents the confusion matrices of all the classification results. A comparison with previous results is shown in Table S5, which indicates our proposed model can solve the complicated hydraulic system diagnosis problems.

*
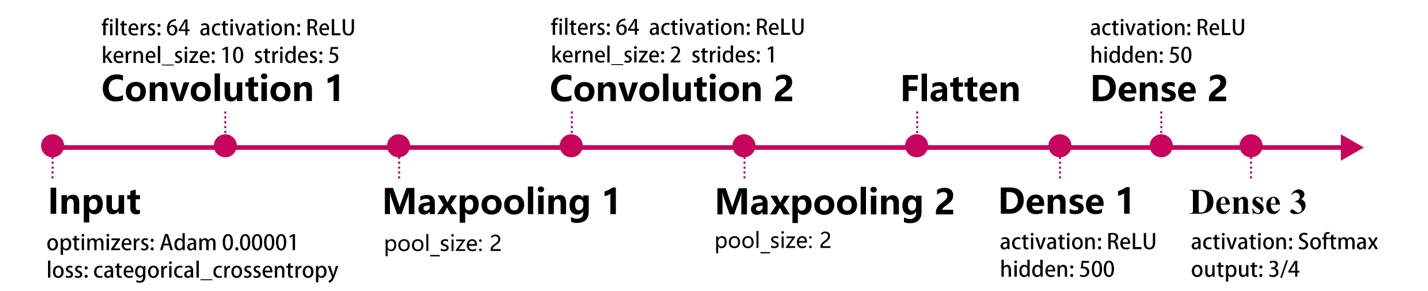
*

Fig. S6. The hyper-parameters in hydraulic system condition classification.


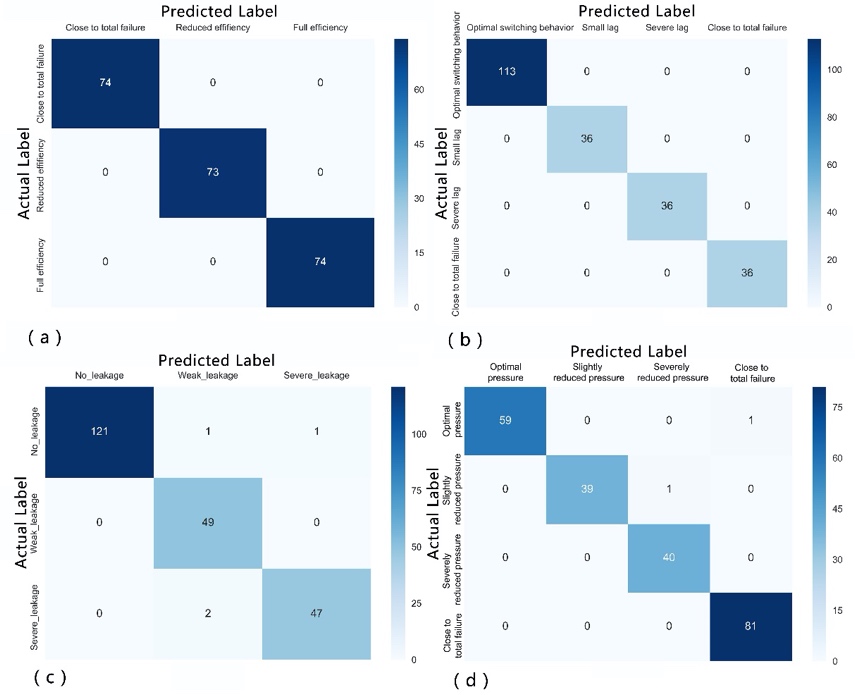


Fig. S7. Confusion matrix of hydraulic system condition classification: (a) three-way classification model for cooler, (b) four-way classification for valve condition, (c) three-way classification model for internal pump leakage, and (d) four-way classification model for hydraulic accumulator.

Table S5: A comparison of the classification results.

|  | Cooler condition  (Three-way task) | Valve condition  (Four-way task) | Internal pump leakage  (Three-way task) | Hydraulic accumulator  (Four-way task) |
| --- | --- | --- | --- | --- |
| **Proposed model** | **100%** | **100%** | **98.19%** | **99.10%** |
| Helwig *et al.* [15] | 100% | 100% | 98.00% | 90.40% |

*Robustness analysis:* The additive Gaussian noise whose power $P$ varies from 0% to 1% of each original signal power $P_{0}$ are used to verify the robustness of the proposed method. The comparisons between the raw data curve and the data curve with 0.001%, 0.1%, and 1% noise are shown in Fig. S8 (a-c). Different from other cases in this paper, the noise ratio is small but has larger effects on magnitude, and make the accuracies drastically change in these diagnosis models. Similarly, 90% of samples are used for training, while the rest 10% of samples are used for test. As the power of noise increases, the classification accuracies of four models decrease (see in Fig. S8 (d)). From Fig. S8 (d), we could find the accuracies surpass 60% in the four models when the powers of noises are smaller than 0.1%.


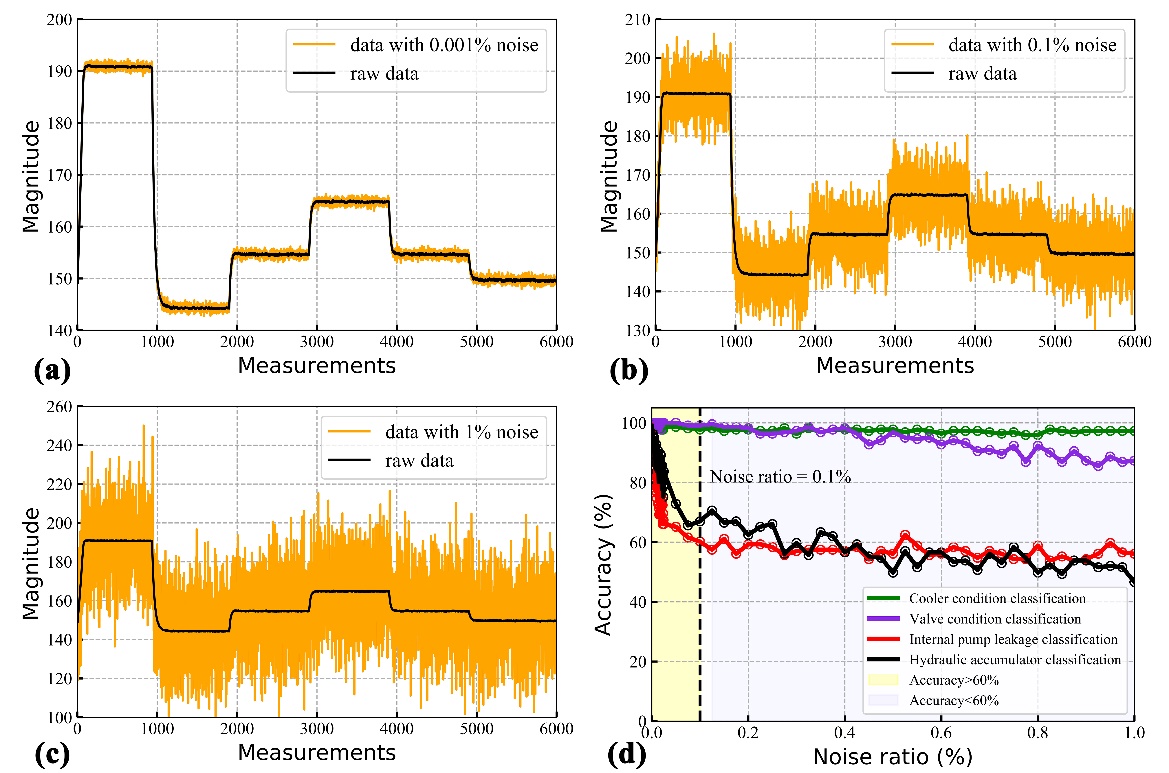


Fig. S8. Comparisons between raw data and (a) raw signal plus additive Gaussian noise whose P=0.001% $\cdot P_{0}$; (b) raw signal plus additive Gaussian noise whose P=0.1% $\cdot P_{0}$; (c) raw signal plus additive Gaussian noise whose P=1% $\cdot P_{0}$. (d)The curves of accuracies change with noise ratio for three classification models.

# Case 3: Tool broken classification

*Problem formulations:* Broken tools can lead to an interruption of production and damage the work piece surface. This commonly happens when heavy cutting force occurs in manufacturing. Conventionally, the discovery of the tool broken depends on human inspection. Here we apply the proposed data-driven CNN model to discover the tool broken in manufacturing process. We apply a CNN model to detect the tool broken during the machining process.

*Introduction and properties of data set:* Tool broken data in this case is provided by our industrial partner with permission in the field of vehicle manufacturing. Properties of this data set include the following:

- This data set contains 28 samples generated by 14 tool wear processes. Each process contains a normal sample and a tool broken sample, and every tool wear process uses the same type of tool.
- Current sensors are used to record the three-phase currents (denoted by A phase, B phase, and C phase), while A phase current is used for tool broken detection.
- The rotational speed of spindle is 3500 rpm and the sampling frequency is 10 kHz.
- Tools are used to process turbofans.
- Based on A phase current data, we apply CNN, which can readily classify the data into two classes: one class corresponding to normal processing and another class corresponding to the tool broken.
- Each sample contains 6000 measurements.

*Data pre-processing:* To evaluate model performance, the obtained entire data set is divided into training set with 22 samples and test set with 6 samples.


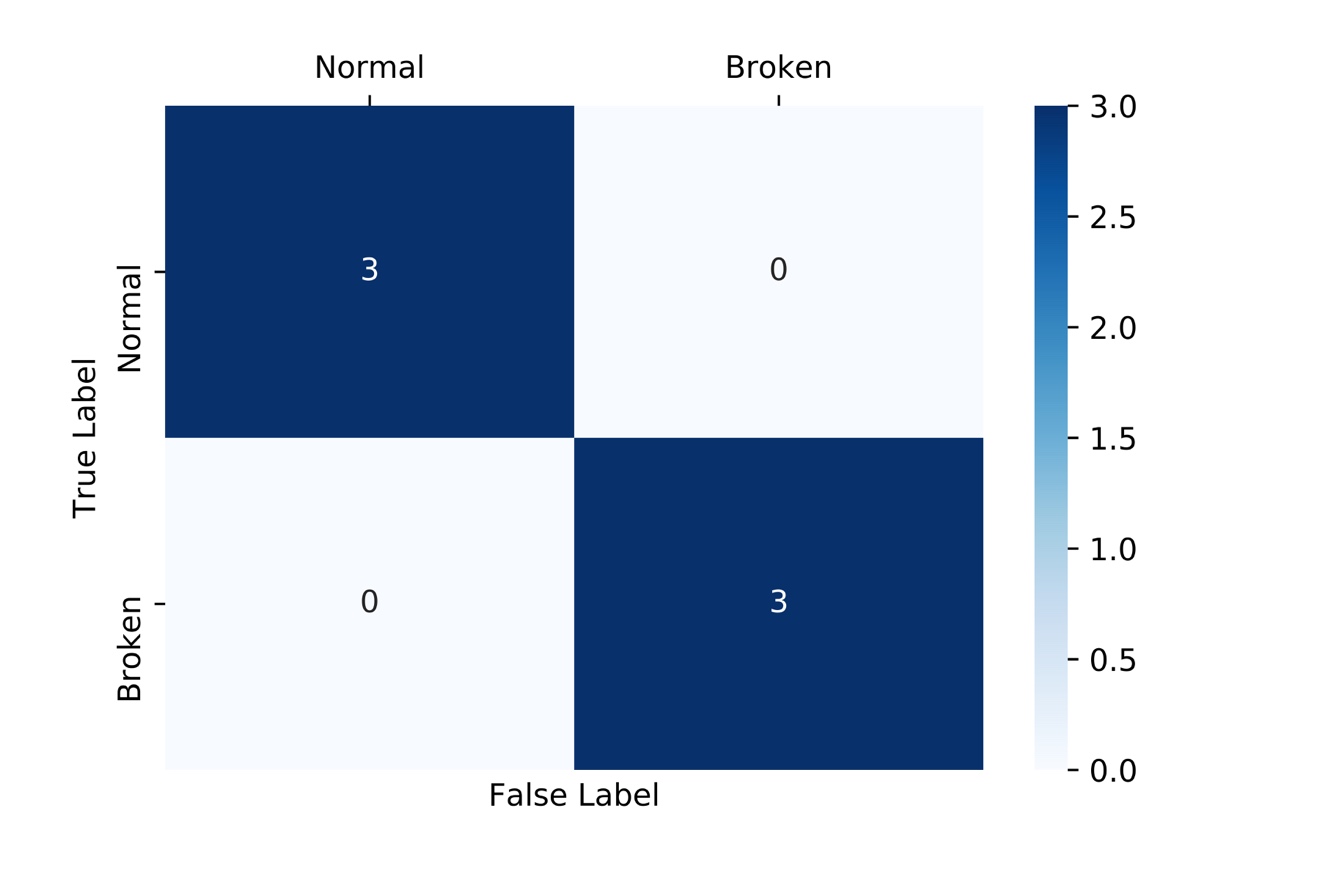
*Prediction results:* We fit the training data and testify the test data with a model with a 3-layer CNN including two fully connected layers. A high accuracy result of 100% is achieved, showing its ability to identify the tool condition from a cluttered data. Fig. S9 displays the confusion matrix of the classification results. Detailed tuning parameters in loss function, optimizer, and layer parameters can refer to the model parameters and structure in Fig. S10.

Fig. S9. Classification confusion matrix of tool broken classification task.


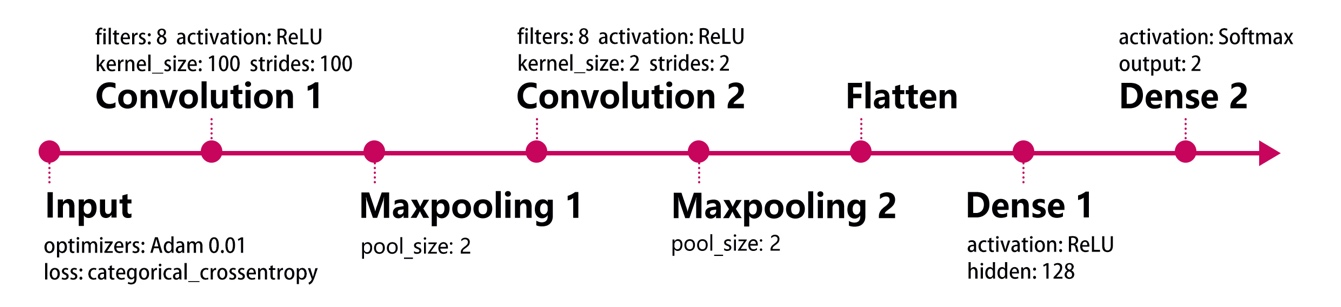


Fig. S10. CNN structure and parameters of tool broken classification task.

*Robustness analysis:* To verify the robustness of the proposed approach, additive Gaussian noise, whose power $P$ varies from 0% to 600% of each original signal power $P_{0}$, is added to each sample. Fig. S11 (a-c) show the noisy data with different power (10%, 200%, and 600%), where the noise will extremely affect the power of the original data. Fig. S11 (d) expresses the classification accuracy changes with noise ratio, where the accuracy is equal to 100% when the noise ratio is smaller than or equal to 120%.


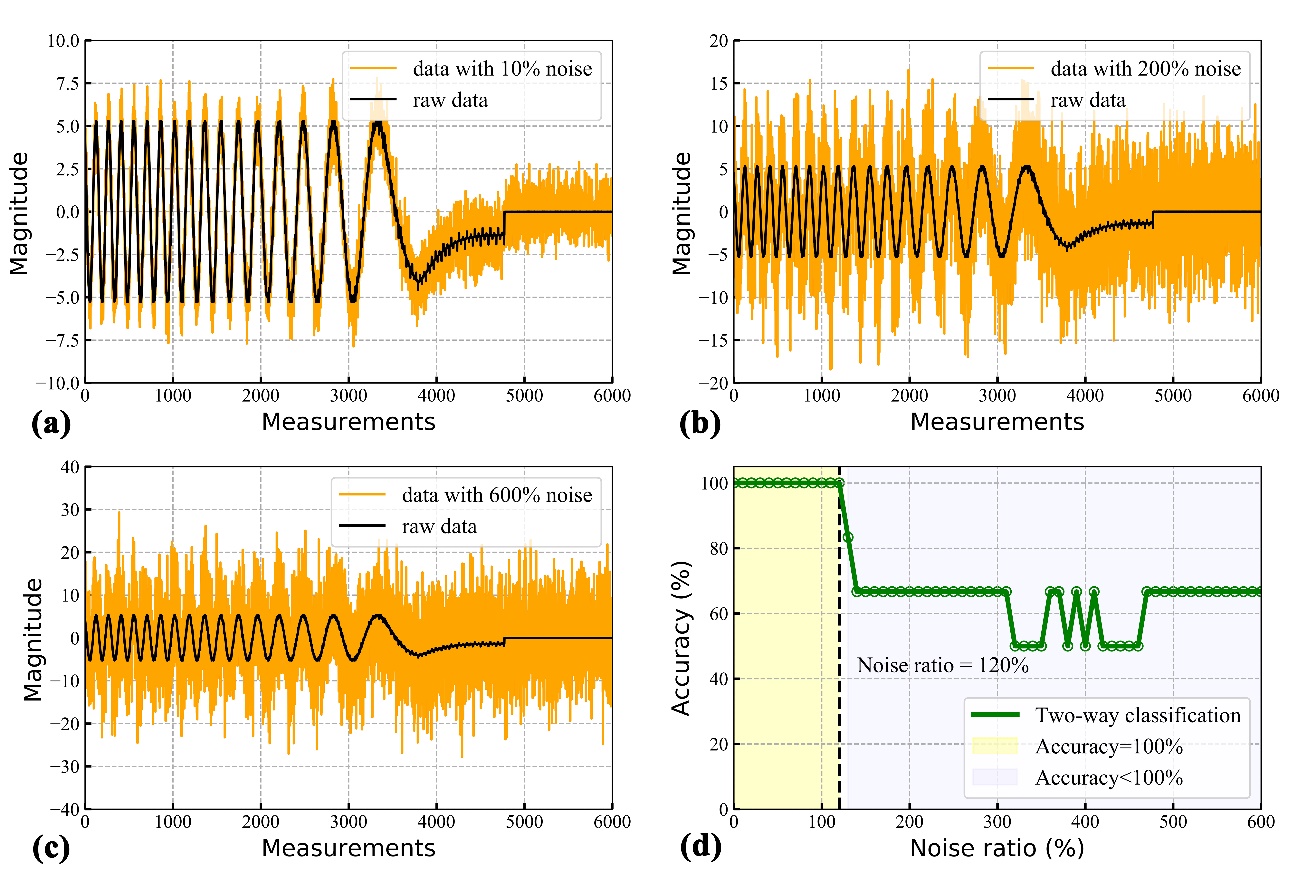


Fig. S11. Comparisons between raw data and (a) raw signal plus additive Gaussian noise whose P=10% $\cdot P_{0}$; (b) raw signal plus additive Gaussian noise whose P=200% $\cdot P_{0}$; (c) raw signal plus additive Gaussian noise whose P=600% $\cdot P_{0}$. (d) The curve of accuracy with noise ratio increasing for the classification model.

# Case 4: Rolling bearing fault classification

*Problem formulations:* In view of the importance of rotating machinery to bearing failures, we also collected condition data from experiments that are intended to test whether CNN models are versatile in diagnosing bearing fault types.

*Introduction and properties of data set:* Experimental data of rolling bearings are collected by our laboratory, collaborated with Jiangnan University [16]. This data set is similar to the bearing data set in *Case 1* but only containing the bearing of 14-mil fault diameter. Properties of this data set include the following:

- This case utilizes voltage signals from the motor by an accelerometer sensor (sampling frequency is 50 kHz) as the inputs for CNN model.
- Four conditions of voltage signals are collected, namely normal condition, faulty condition with fault at rolling elements, faulty condition with fault at outer race, and faulty condition with fault at inner race.
- Three bearings are tested for each operational condition.
- We conduct a binary classification (normal and faulty condition) task and a four-way classification (normal and three faulty conditions at different places of the bearing) task.

*Data pre-processing:* The experiment provides twelve samples for classification. Each faulty sample and normal sample contain 500,500 and 15,001,500 measurements, respectively. To obtain a constant sample length, firstly, we crop out the first 500,000 measurements of the faulty signal and 15,000,000 measurements of the normal signal. Then, similar to *Case 1*, we divide the samples to make each sample have 10,000 measurements consistently. The detailed treatment is listed in Table S6. We obtained 900 samples in total, that is to say, 450 normal samples and 150 samples for each faulty condition. After above pre-processing, we randomly split the entire data into two parts: 90% for training and 10% for test.

Table S6: Original data and pre-processed data in our experiment data set.

|  | Original data | | Pre-processed data | |
| --- | --- | --- | --- | --- |
|  | Measurements | Sample Numbers | Measurements | Numbers |
| Normal Signal | 15,000,000 | 3 | 10,000 | 450 |
| Inner race fault signal | 500,000 | 3 | 10,000 | 150 |
| Outer race fault signal | 500,000 | 3 | 10,000 | 150 |
| Rolling elements signal | 500,000 | 3 | 10,000 | 150 |

*
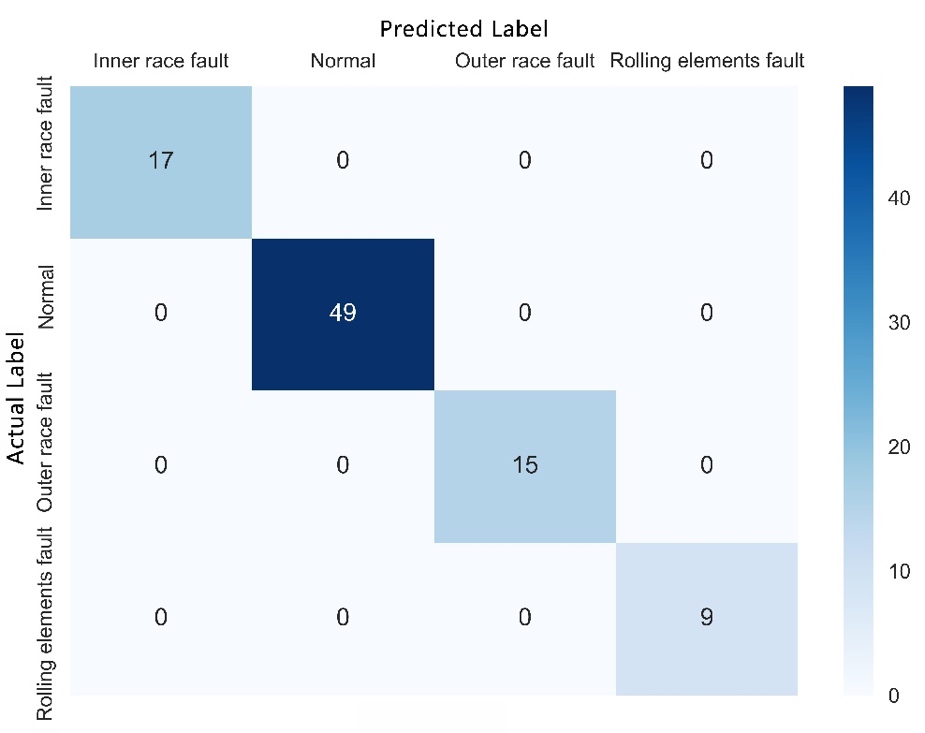
Prediction results:* We train the CNN model with 810 pieces of training data and then use 90 pieces of test data for model verification, while we obtain a high accuracy of 100% (90/90), which indicates the proposed CNN framework has great versatility and has a significance for the future bearing fault diagnosis. Fig. S12 is the confusion matrix of the classification results. Detailed tuning parameters of our proposed model are shown in Fig. S13.

Fig. S12. Confusion matrix of rolling bearing fault classification.


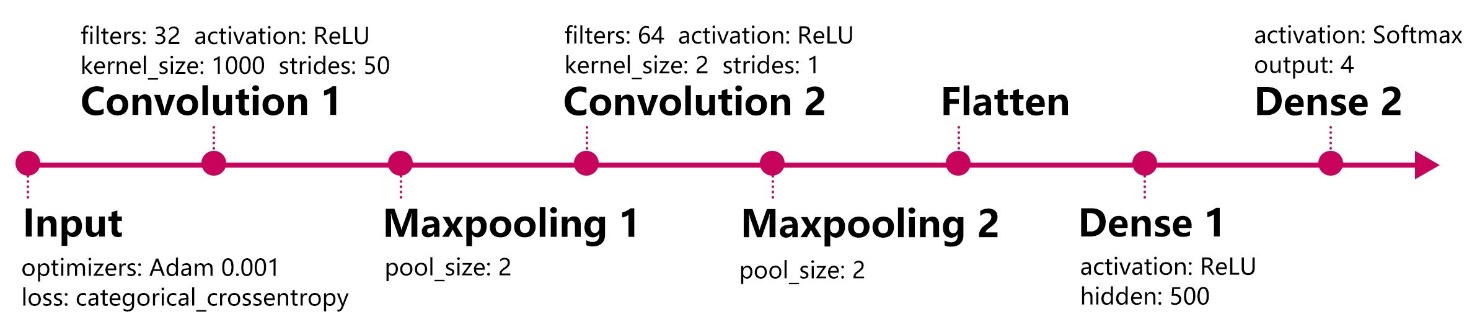


Fig. S13. CNN structure and parameters in rolling bearing data set.

*Robustness analysis:* To verify the robustness of the proposed approach, additive Gaussian noise, whose power $P$ varies from 0% to 200% of each original signal power $P_{0}$, is added to each sample. Fig. S14 (a-c) describe the comparisons among raw data and raw data plus additional noise (20%, 100%, and 200%), which shows that the noise will largely increase the magnitude of the raw data, possibly leading to the misclassification in this task. Fig. S14 (d) shows the accuracy decreases as the noise ratio increases, where the accuracy is more than 97% when the noise ratio is smaller than or equal to 20%.


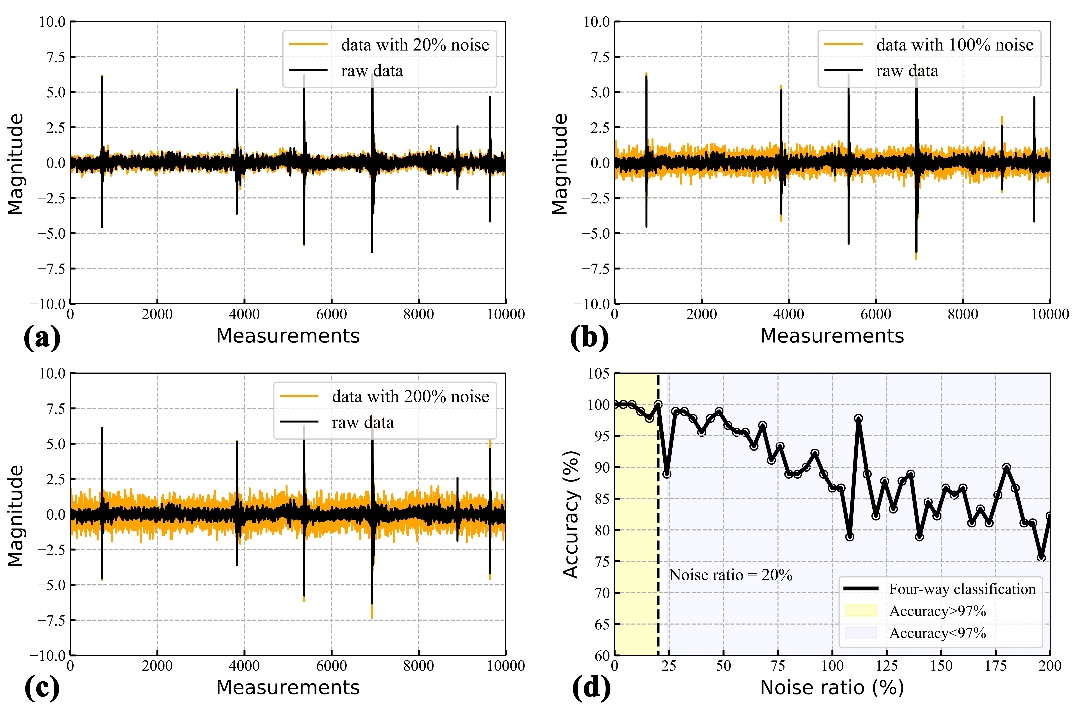


Fig. S14. Comparisons between raw data and (a) raw signal plus additive Gaussian noise whose P=20% $\cdot P_{0}$; (b) raw signal plus additive Gaussian noise whose P=100% $\cdot P_{0}$; (c) raw signal plus additive Gaussian noise whose P=200% $\cdot P_{0}$. (d) The curves of accuracies change with noise ratio for the classification model.

|  | Random subsets | Independent sequence | Contiguous block | | | | |
| --- | --- | --- | --- | --- | --- | --- | --- |
|  |  |  | 10% test | 20% test | 30% test | 40% test | 50% test |
| Four-way classification | 100% (90/90) | 98.67% (296/300) | 100% (90/90) | 96.67% (174/180) | 96.30% (260/270) | 83.33% (300/360) | 80.89% (364/450) |

*Cross validation:* Except the random subsets method, we also employ contiguous block and independent sequence methods of cross-validation to eliminate the time dependency of training samples and test samples, hence to verify the effectiveness of the model. We summarize the results of different cross-validation methods in Table S7.

Table S7: The results of three cross-validation methods for bearing data.

# Case 5: Airplane girder fault classification

*Problem formulations:* As the main support structure of an airplane, the girder has many bolts on it for fixing, and the tightness of bolts will affect the stability of the girder. An unstable girder will reduce the stiffness and the stability of the airplane and increase the risk of the potential danger.

*Introduction and properties of data set:* The girder simulation data [16] is provided by our collaborator from [Nanjing University of Aeronautics and Astronautics](https://f.glgooo.top/citations?view_op=view_org&org=8432782691408189622&hl=zh-CN&oi=io) with permission. The experimental diagram is shown in Fig. S15 (b). Properties of this data set are summarized as follows:

- The experiment utilizes two piezoelectric sensors with a fixed sampling rate at 10 MHz, one for excitation and the other for measuring signals. The distance between the two sensors is 120 mm.
- The experimental data was obtained by artificially controlling the level of the injury and the loosening of the bolt to research the characteristic of the girder and, through the Lamb wave corresponding signal changes, understanding the condition of the girder. The description of this data set is provided in Table S8.
- According to different injury/damage levels and the bolt loosening locations as shown in Table S8, two types of classification tasks can be proposed. First is a five-way classification (in terms of damage-size) for normal condition and four faulty conditions with different damage sizes (i.e., no damage, 4 mm, 8 mm, 12 mm, and 16 mm damage shown in the first row in Table S8). The second task is a four-way classification (in terms of bolt-loose) according to the location of the bolt loosening while ignoring the level of damage (i.e., no bolt loose and bolt loose of no. 2, 4, and 6 shown in the first column in Table S8). The size of each data set is 60$\times$10,000 (60 samples and 10,000 measurements in each sample): thus, each damage-size condition has 240 samples, while 300 samples exist in each bolt-loose condition.

Table S8: The description of airplane girder simulation data set.

|  | **No**  **Damage** | **4 mm Damage** | **8 mm Damage** | **12 mm Damage** | **16 mm Damage** | **Sum** |
| --- | --- | --- | --- | --- | --- | --- |
| **No** **Bolt** | 60$\times$10,000 | 60$\times$10,000 | 60$\times$10,000 | 60$\times$10,000 | 60$\times$10,000 | 300$\times$10,000 |
| **Bolt no.2** | 60$\times$10,000 | 60$\times$10,000 | 60$\times$10,000 | 60$\times$10,000 | 60$\times$10,000 | 300$\times$10,000 |
| **Bolt** **no.4** | 60$\times$10,000 | 60$\times$10,000 | 60$\times$10,000 | 60$\times$10,000 | 60$\times$10,000 | 300$\times$10,000 |
| **Bolt no.6** | 60$\times$10,000 | 60$\times$10,000 | 60$\times$10,000 | 60$\times$10,000 | 60$\times$10,000 | 300$\times$10,000 |
| **Sum** | 240$\times$10,000 | 240$\times$10,000 | 240$\times$10,000 | 240$\times$10,000 | 240$\times$10,000 | 1200$\times$10,000 |

*Data pre-processing:* For the damage-size classification, we have 240 samples for each of five damage conditions, so that in total 1200 samples are used. For the bolt-loose classification, we also have 1200 samples in total with respect to 300 samples for each of the four loose conditions. For both classifications, data are randomly split into 90% for training, and 10% for test.

*Prediction results:* We achieve a high accuracy of 100% in both classifications, which demonstrates that the proposed CNN framework has a great significance of fault diagnosis for airplane girders. A sketch map of the damage-size classification result on test data sets is presented in Fig. S15(a). The bolt-loose classification task performs the similar result. Fig. S16 shows the confusion matrices of the two tasks. Detailed tuning parameters of the damage-size and bolt-loose classifications, such as kernel size and max-pooling size, can refer to the model parameters in Fig. S17.


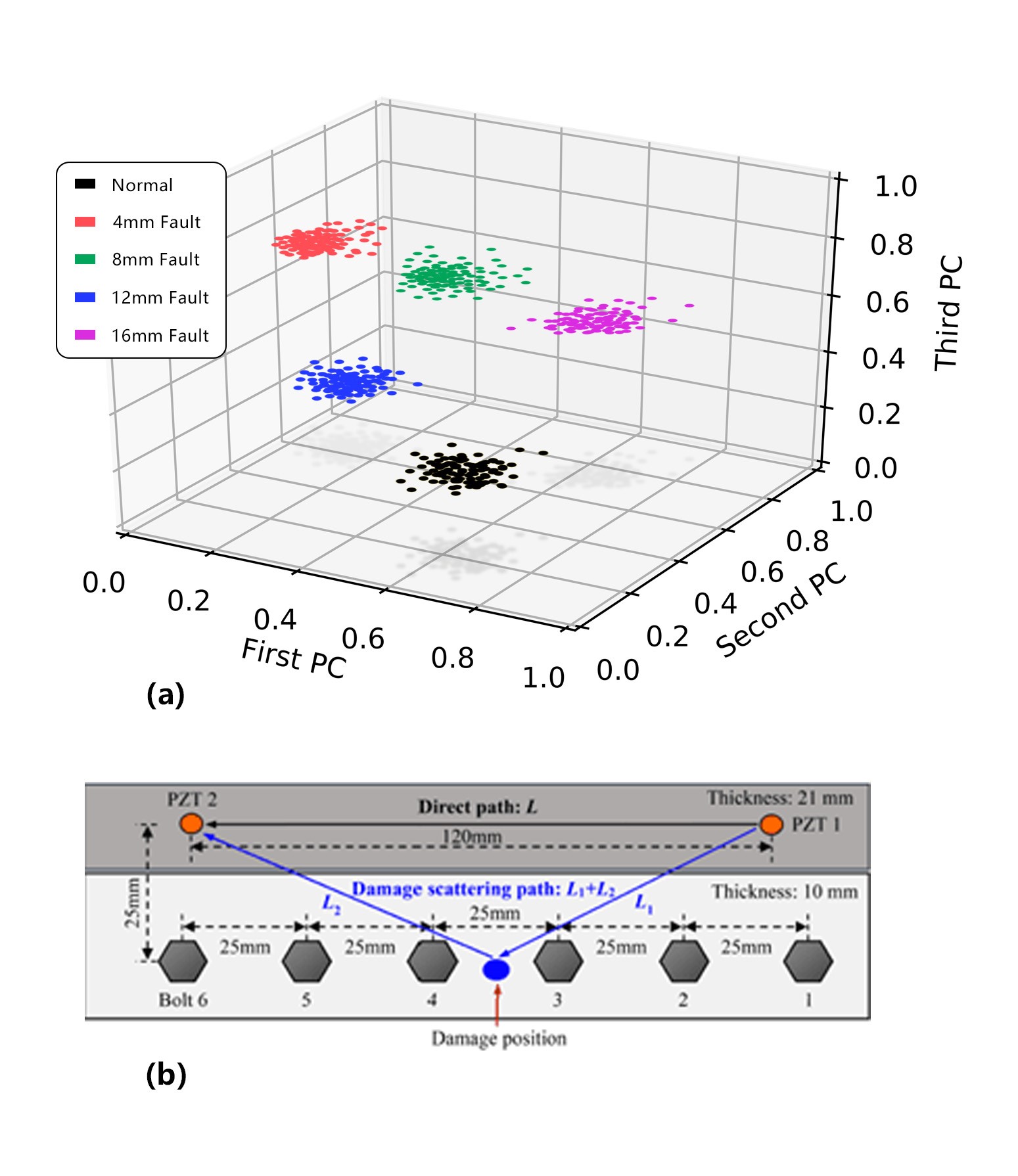


Fig. S15. Airplane girder fault classification. (a) Sketch maps of classification results on test data sets o are shown in three-dimensional coordinate system; (b) The [schematic](file:///E:\%E6%9C%89%E9%81%93%E8%AF%8D%E5%85%B8\Dict\7.5.2.0\resultui\dict\?keyword=schematic)[diagram](file:///E:\%E6%9C%89%E9%81%93%E8%AF%8D%E5%85%B8\Dict\7.5.2.0\resultui\dict\?keyword=diagram) and the damaged location of the experiment.


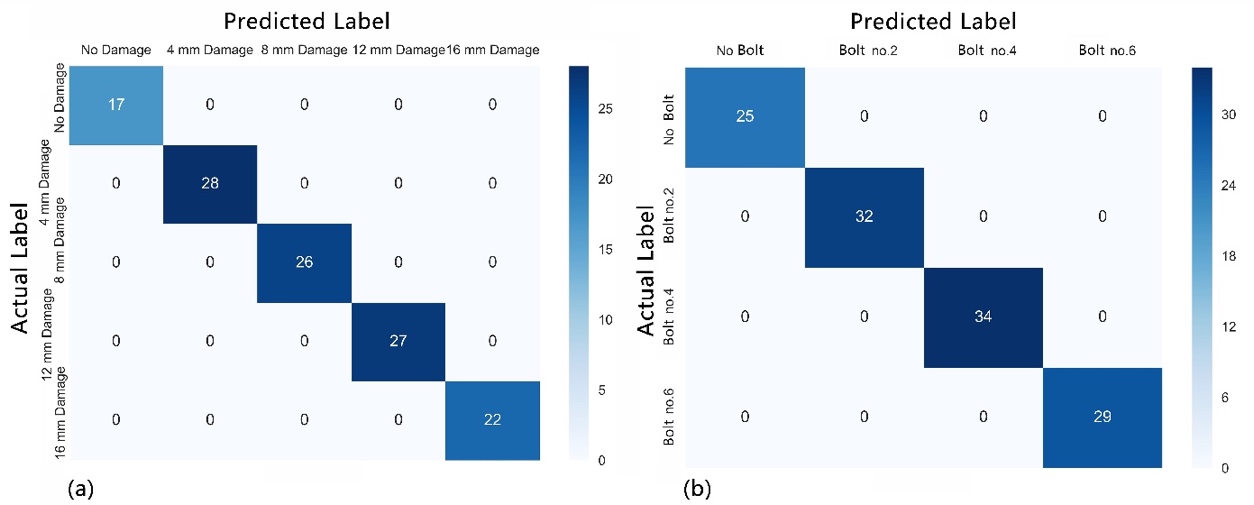


Fig. S16. Confusion matrices of airplane girder fault classification: (a) five-way classification and (b) four-way classification.


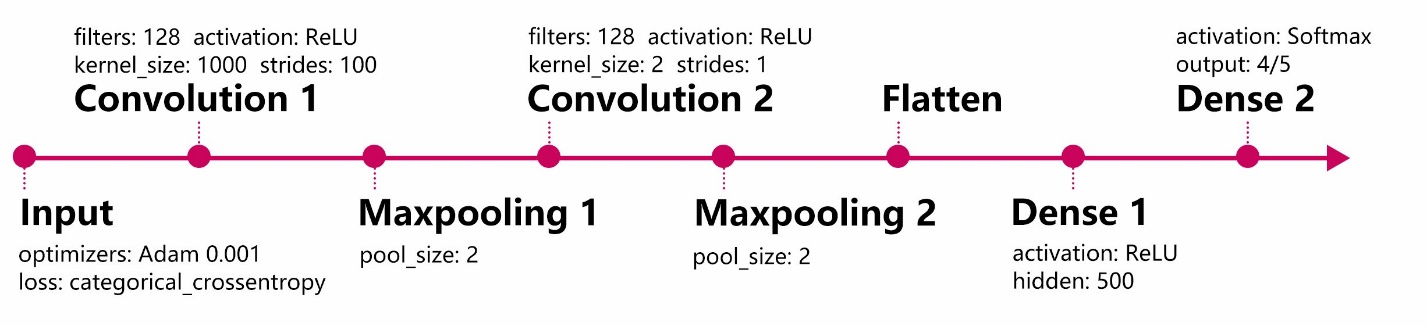


Fig. S17. CNN structure and parameters in airplane girder simulation damage data classification.

*Robustness analysis:* The robustness verification in this case is the same as the previous ways. Additive Gaussian noise, whose power $P$ varies from 0% to 20% of each original signal power $P_{0}$, is employed to verify the robustness of the proposed method for the two diagnosis models. The noisy signals (2%, 10%, and 20% noise) and raw data are shown in Fig. S18 (a-c), where we find the noise could largely affect the magnitude of the original data. Fig. S18 (d) expresses the accuracies for the two classification models varies with noise, where we can find the accuracies are still more than 98% when the noise ratios are smaller than or equal to 2%.


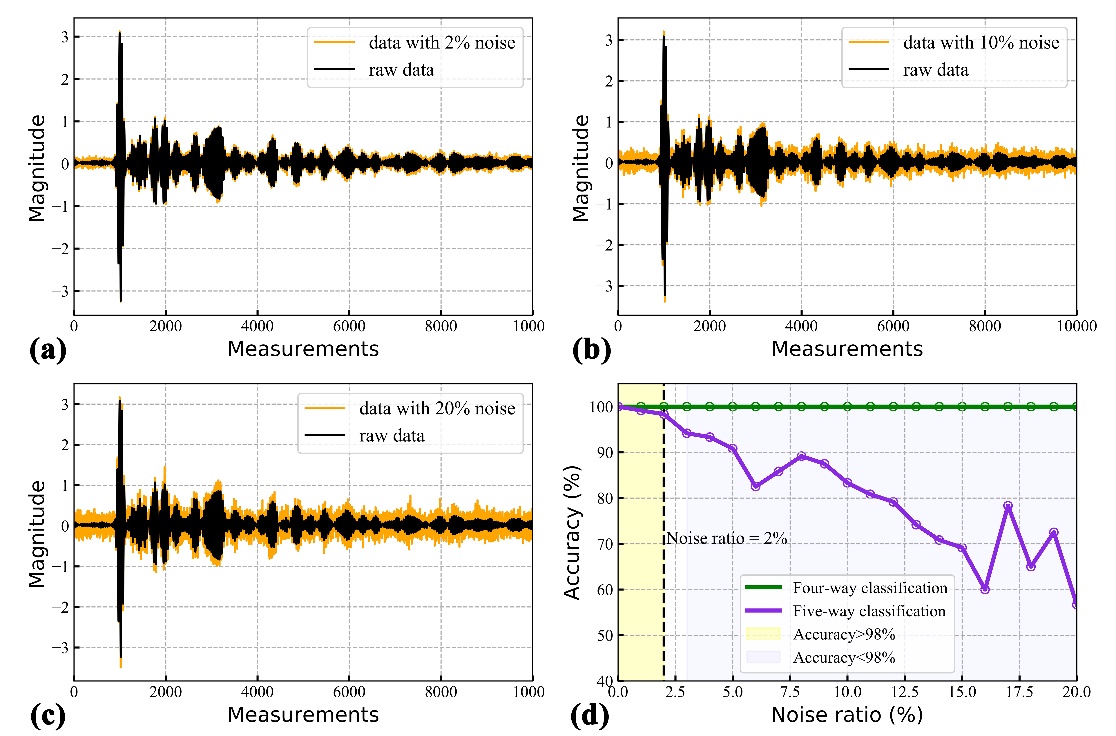


Fig. S18. Comparisons between raw data and (a) raw signal plus additive Gaussian noise whose P=2%$\cdot P_{0}$; (b) raw signal plus additive Gaussian noise whose P=10% $\cdot P_{0}$; (c) raw signal plus additive Gaussian noise whose P=20% $\cdot P_{0}$. (d)The curves of accuracies vary with noise ratio for the classification models.

# Case 6: Process classification

*Problem formulations:* In this case, a high-precision processing of cutter-machined aero-engine compressor blade is considered, while the collected data pertains to the step of blade processing and precision milling.

*Introduction and properties of data set:* The data set used in this case was acquired in our laboratory. Completing one work piece by precision milling is a complicated task that goes through a total of 30 processes. We would like to verify whether the proposed CNN technique can accurately classify the process types. Properties of this data set are summarized as follows:

- The precision milling process in this case is conducted under a real industrial application. Therefore, the whole process experiences much longer time than other simulation models or experimental platforms.
- The experiment for collecting voltage signal using Hall sensors is illustrated in Fig. S19.
- 24 work pieces are inspected with a fixed sampling frequency of 12 kHz. 4 processes of all the work pieces with the most frequent tool changes are selected for classification.
- The data set contains 489 samples, including 120 samples for process one, 123 samples for process two, 120 samples for process three, and 126 samples for process four.

*Data pre-processing:* Similar to *Case 1* with high sampling frequency, each sample of each process contains more than 410,000 measurements. We make use of the first 410,000 measurements of each sample for model input uniformity. Before a classification, we randomly split the entire data set into 90% training set and 10% test set.

*Prediction results:* We used 391 samples for training and the rest for test. After fine tuning the parameters of CNN model, we achieved a high accuracy of 95.92% (94/98) in process classification, which indicates that the CNN model in question can function as a universal method for classifying manufacturing data set. Fig. S20 presents the confusion matrix of the classification results. Detailed tuning parameters, such as kernel size and max-pooling size, can refer to the model parameters in Fig. S21.


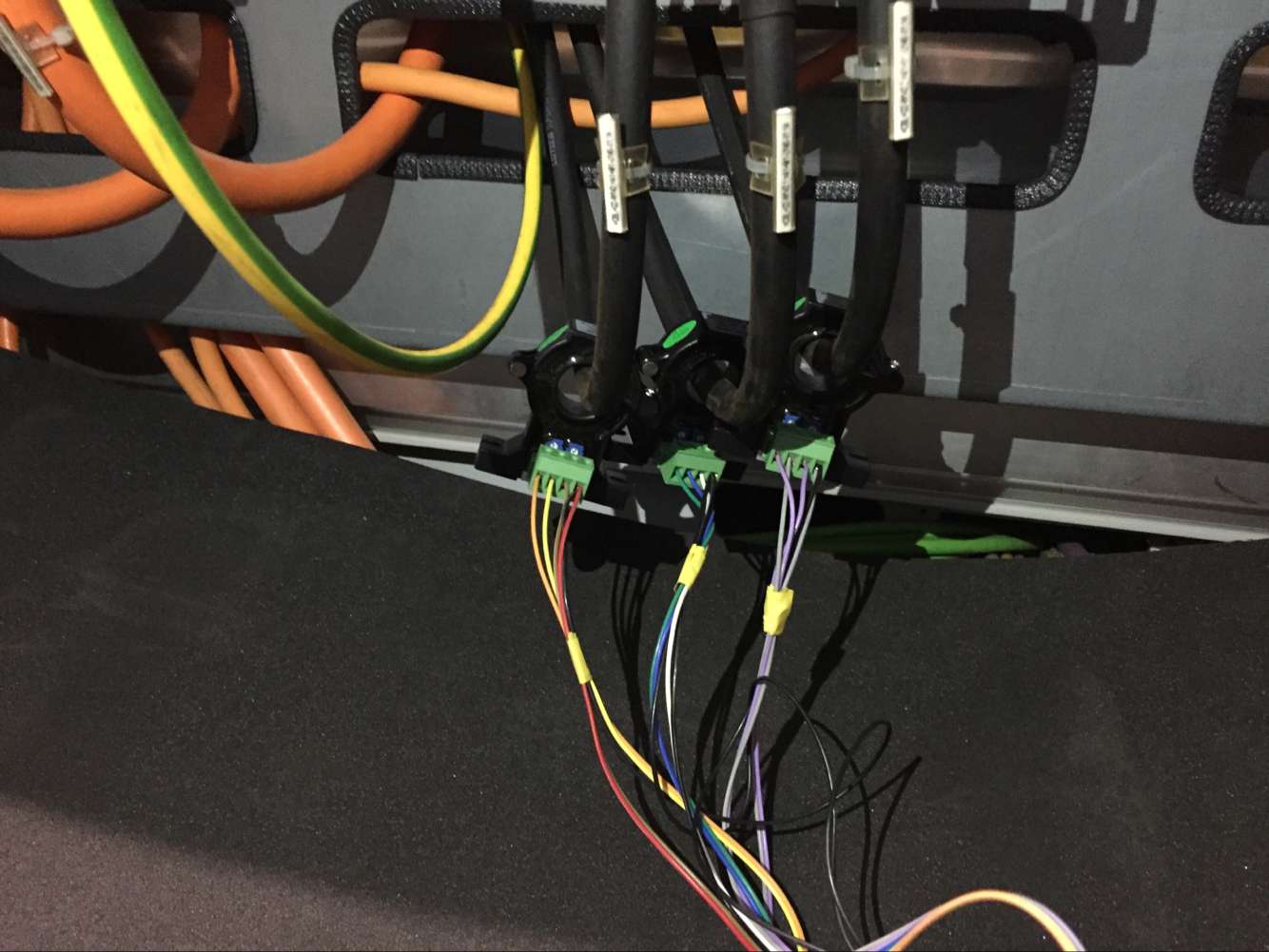


Fig. S19. The experiment for collecting voltage signal.


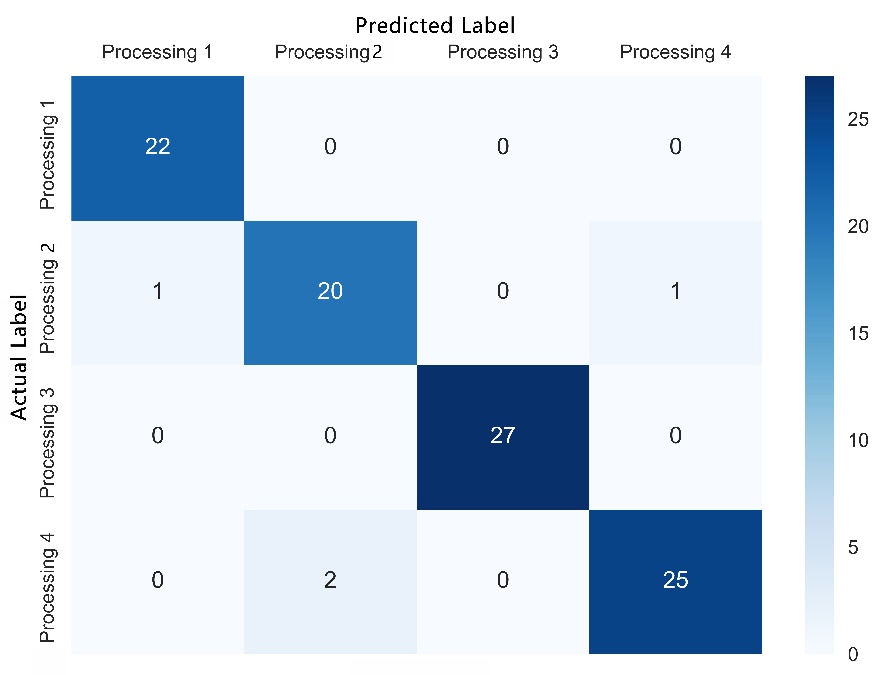
Fig. S20. Confusion matrix of process data set classification.


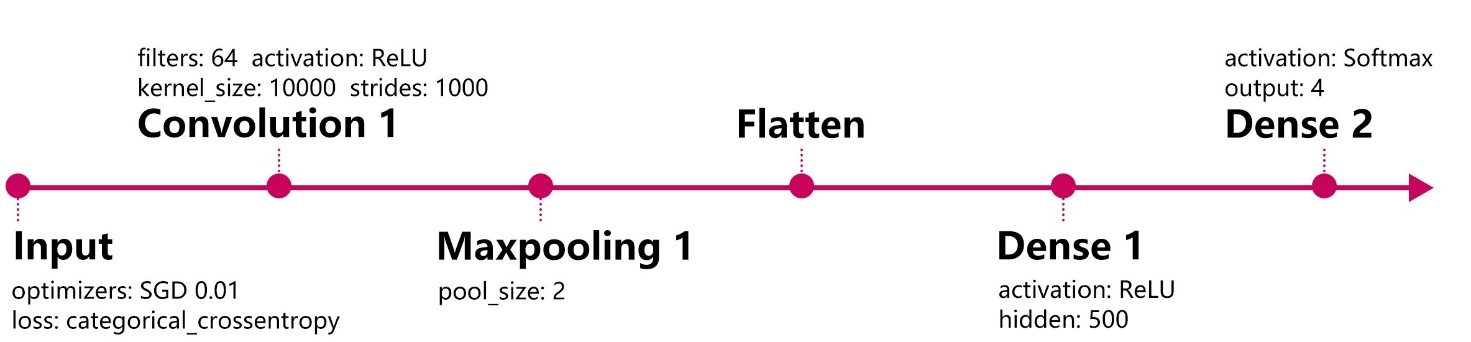


Fig. S21. CNN structure and parameters in process data set classification.

*Robustness analysis:* The additive Gaussian noise, whose power $P$ ranges from 0% to 150% of each original signal $P_{0}$, is added to each raw signal. Fig. S22 (a-c) show the noisy data with different power (20%, 80%, and 150%), where the noise will extremely affect the power of the original data. Fig. S22 (d) expresses the classification accuracy changes with noise ratio increases, where the accuracy is more than 90% when the noise ratio is smaller than or equal to 40%.


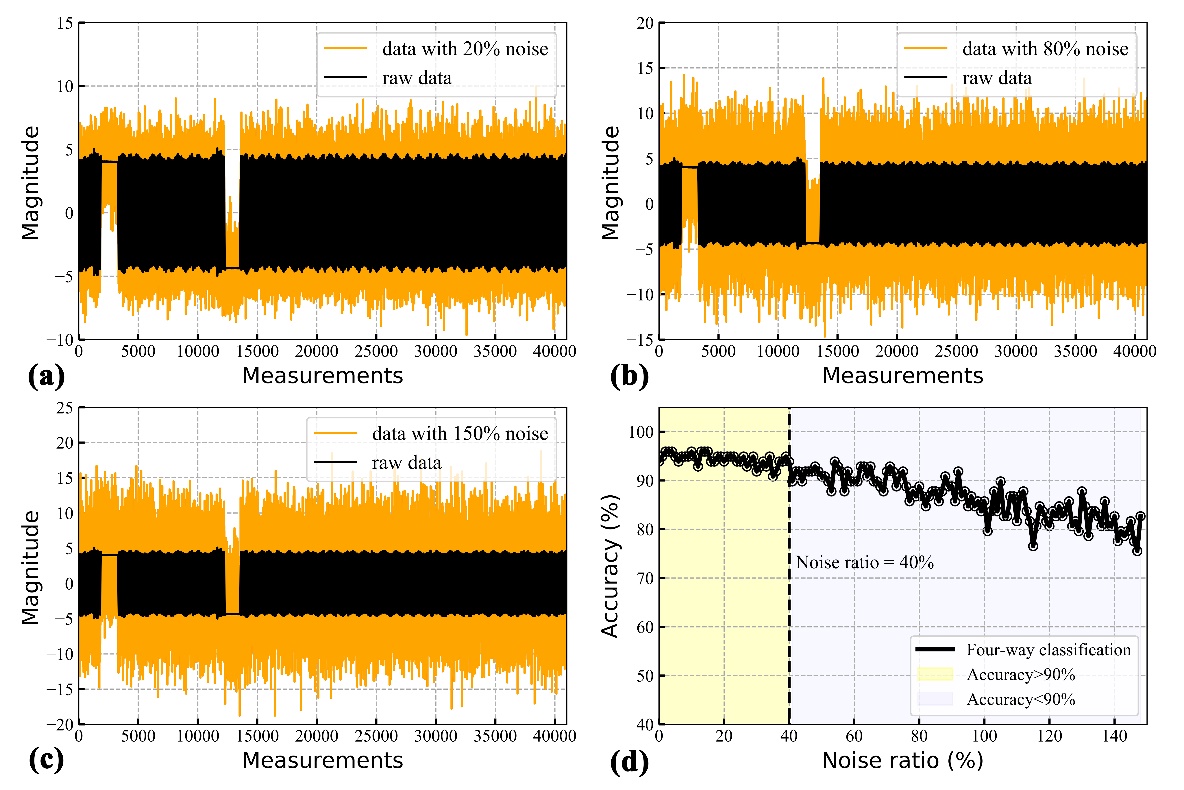


Fig. S22. Comparisons between raw data and (a) raw signal plus additive Gaussian noise whose P=20% $\cdot P_{0}$; (b) raw signal plus additive Gaussian noise whose P=80% $\cdot P_{0}$; (c) raw signal plus additive Gaussian noise whose P=150% $\cdot P_{0}$. (d) The curve of accuracy changes with noise ratio for the classification model.

# Case 7: Anomaly detection of gearbox


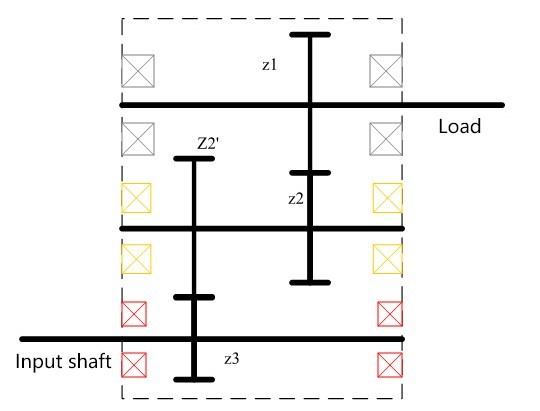
*Problem formulations:* Gearbox is one of the most common mechanical structures, consisting of different speed gears and the gear train, which allow speed and torque conversions by different gearbox ratios. Driving with a heavy load on the output shaft side may cause serious gear wear or cause the teeth broken. The schematic diagram of the experimental gearbox is presented in Fig. S23. The input shaft is a high-speed shaft and the output shaft is a low-speed shaft that carries the load from other devices. The middle shaft is a transition shaft.

Fig. S23. Schematic diagram of the experimental gearbox. High-speed gear, z3, and low-speed gear, z1, carry the input shaft and the output shaft, respectively. Z2’ and z2 are the intermediate gears (middle gears) mesh with the toothed part of input gear and output gear to transmit torque.

*Introduction and properties of data set:* Gearbox signals are inspected on an experimental platform, which has been discussed in [17]*.* This experiment artificially destroys the gear. The following characteristics can be observed:

- Two accelerometers are designed to collect the vertical and horizontal vibration signals, and a current sensor is used to collect the current signal. For data analysis, we use only the vertical vibration signal as the input of our model.
- Four operation conditions are inspected: 1) one tooth broke in high-speed shaft side; 2) the high-speed shaft breaks another tooth; 3) the high-speed shaft gear breaks one tooth. In the meantime, the middle shaft gear breaks one tooth, which meshes with the high-speed gear; and 4) all of the gears are working in normal condition.
- Each condition has about 8 samples, and measurements in each sample are varied from 2,046 to 200,728.
- We transform the problem of gearbox fault diagnosis into supervised classification problems. Here we can construct a binary classification (normal and failure) and four-way classification (normal and three types of broken teeth) tasks.

*Data pre-processing:* To increase the number of samples, similar to the CWRU bearing data, we assume that the signal after the tooth broken is a stationary process. We divide each sample data to ensure that each sample has 6000 measurements: samples having fewer than 6000 measurements are removed. By this means, 715, 294, 183 and 662 samples are generated in four conditions. For test purpose, we randomly split the entire data set into 80% (training), and 20% (test).

*Prediction results:* For the binary classification (normal and failure), CNN achieves 100% (371 of 371 test samples) accuracy. For the four-way classification (normal and three types of broken teeth), CNN still has 99.46% (369 of 371 test samples) accuracy. Detailed tuning parameters, such as kernel size and max-pooling size, can refer to the model parameters in Fig. S24. Confusion matrices of the classification results are shown in Fig. S25.


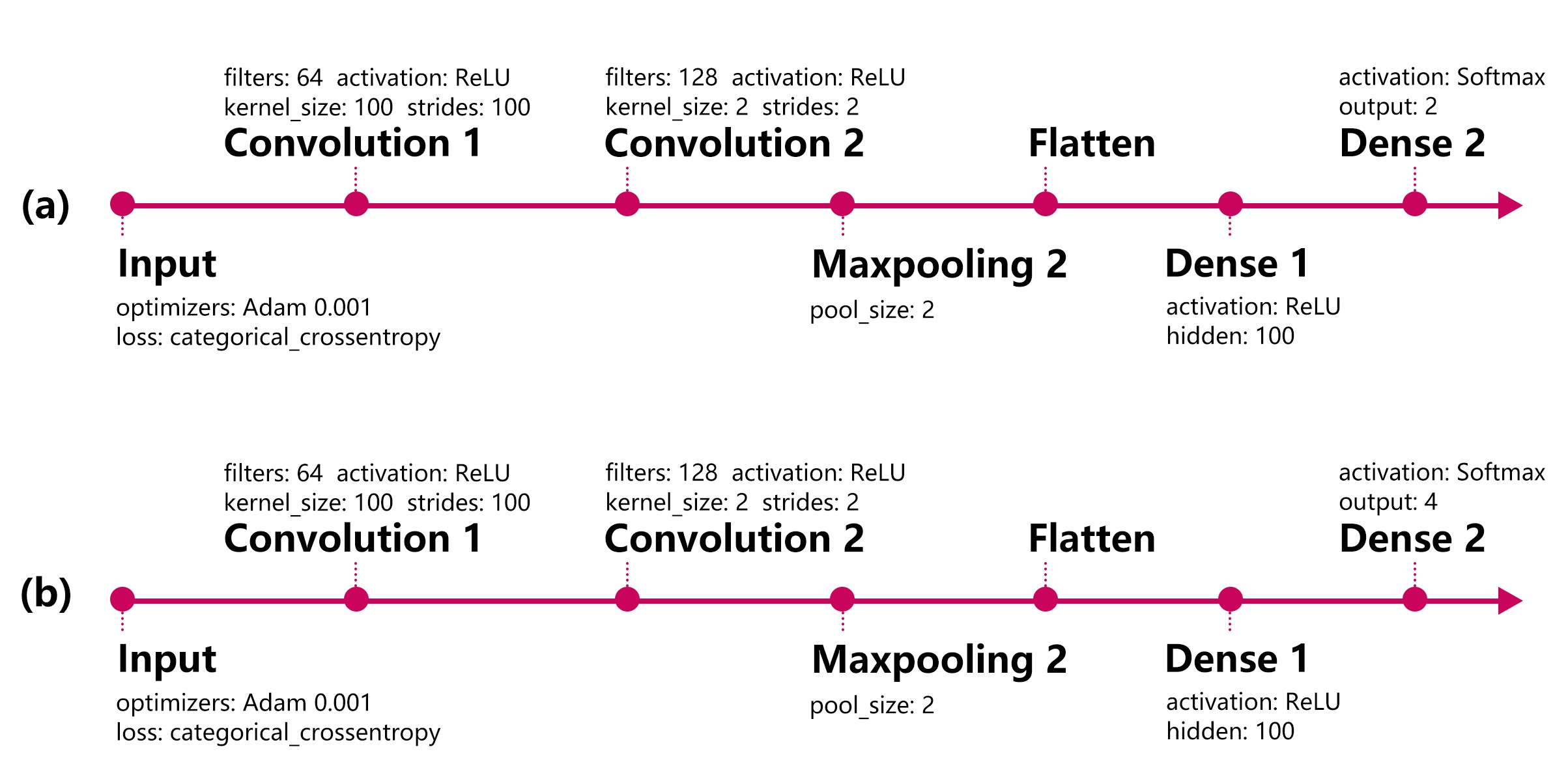


Fig. S24. Binary and four-way classification parameters in gearbox fault classification. (a) Parameters in binary classification. (b) Parameters in four-way classification.


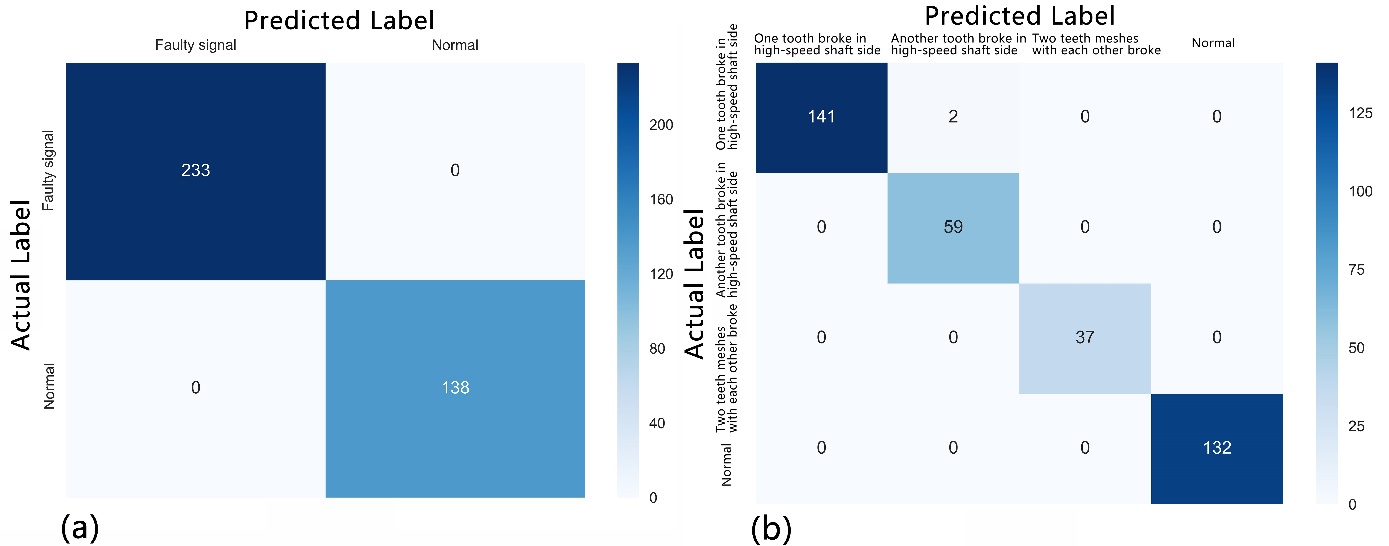


Fig. S25. Confusion matrix of gearbox fault classification: (a) binary classification and (b) four-way classification.

*Robustness analysis:* The additive Gaussian noise, whose power $P$ ranges from 0% to 500% of each original signal $P_{0}$, is added to each raw signal. 50% noise, 200% noise, and 500% noise are expressed in Fig. S26 (a-c), respectively. Fig. S26 (d) shows the accuracies of the two models varies with the noise ratio, where the accuracies are more than 97% when the noise ratios are smaller than or equal to 200%.


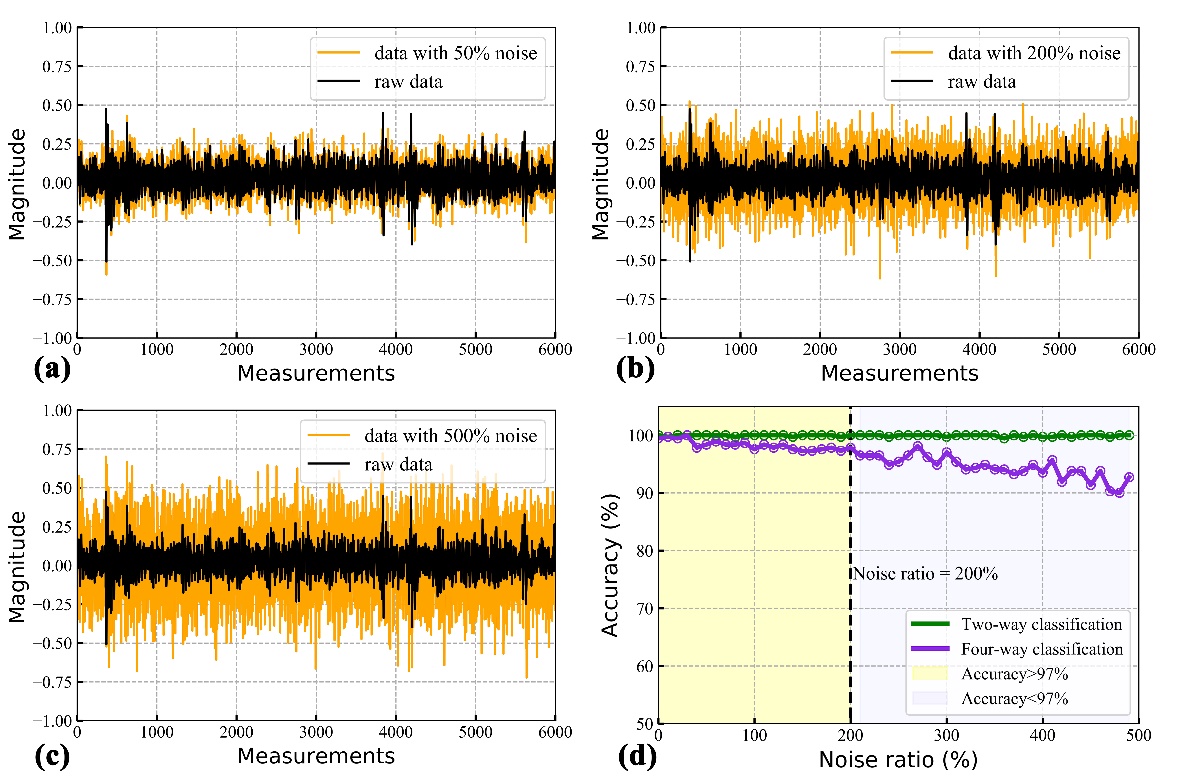


Fig. S26. Comparisons between raw data and (a) raw signal plus additive Gaussian noise whose P=50% $\cdot P_{0}$; (b) raw signal plus additive Gaussian noise whose P=200% $\cdot P_{0}$; (c) raw signal plus additive Gaussian noise whose P=500% $\cdot P_{0}$. (d) The curves of accuracies change with noise ratio for the classification models.

*Cross validation:* Except the random subsets method, we also employ contiguous block and independent sequence methods of cross-validation to eliminate the time dependency of training samples and test samples, hence to verify the effectiveness of the model. We summarize the results of different cross-validation methods in Table S9.

Table S9: The results of three cross-validation methods for gearbox data.

|  | Random subsets | Independent sequence | Contiguous block | | | | |
| --- | --- | --- | --- | --- | --- | --- | --- |
|  |  |  | 10% test | 20% test | 30% test | 40% test | 50% test |
| Two-way  classification | 100% (371/371) | 100% (184/184) | 100% (229/229) | 100%  (404/404) | 100%  (580/580) | 100% (781/781) | 100%  (951/951) |
| Four-way  classification | 99.46% (369/371)) | 100% (184/184) | 100% (229/229) | 100% (404/404) | 100% (580/580) | 100% (781/781) | 99.58%  (947/951) |

# Case 8: Prediction of tool wear value with regression models

*Problem formulations:* Machine tools play an important role in achieving sustainable manufacturing [18] given its strong relation to production quality. The consumption of machine tools is approximately 100 billion USD per year globally [19]. Conventionally, manufacturers change tools at a prefixed usage time while processing. However, this standardized changing time can lead to two types of unexpected results: some tools can continue to operate for longer than the prefixed usage time, in which case the changing policy can lead to a waste of resources; while other tools do not last so long, in which case the policy can lead to a high scrap rate. Therefore, we propose a real-time monitoring scheme for the machine tools based on the measurement data.

*Introduction and properties of data set:* Here, we perform flank wear (denoted as VB) prediction of a milling machine by applying the proposed CNN method, to monitor the health condition of the machine tools using a benchmark data set. This data set is provided by the BEST lab at UC Berkeley and shared online by America National Aeronautics and Space Administration (NASA) data center [20]. Properties of this data set include the following:

- Different types of sensors were used to record data samples and monitor the tool wear stages, including two acoustic emission sensors attached on the table and the spindle, two vibration sensors, and two current (i.e., DC and AC) sensors. To increase model performance, our model input consists of all 6 kinds of sensory signals.
- 16 pieces of flanks under 16 different operating conditions were measured under a cutting speed of 200 m/min, in terms of the different depth of cut, feed, and material. They are summarized in Table S10.
- The root mean square (RMS) values of the signals are measured with a fixed frequency of 250 Hz of each flank. The flank wear is observed by using a microscope and each experiment is terminated when the flank wear reached its failure threshold.

Characteristics of the acquisition data are observed as follows: 1) the time intervals between different samples are not consistent; 2) some flanks stop working in good operating conditions while some other flanks are measured beyond their wear limits; and 3) there exists multiple missing data. In our CNN approach, the inconsistency of interval time does not affect the prediction because the proposed model is an end-to-end process (signals to VB). Meanwhile, the proposed model can predict the VB without the constraint of wear limits as long as the machine tool is working. However, missing data have also been considered in the pre-processing.

Table S10: The structure of NASA machine tool data.

| Condition | Depth of Cut | Feed | Material | Condition | Depth of Cut | Feed | Material |
| --- | --- | --- | --- | --- | --- | --- | --- |
| 1 | 1.5 | 0.5 | 1 - cast iron | 9 | 1.5 | 0.5 | 1 - cast iron |
| 2 | 0.75 | 0.5 | 1 - cast iron | 10 | 1.5 | 0.25 | 1 - cast iron |
| 3 | 0.75 | 0.25 | 1 - cast iron | 11 | 0.75 | 0.25 | 1 - cast iron |
| 4 | 1.5 | 0.25 | 1 - cast iron | 12 | 0.75 | 0.5 | 1 - cast iron |
| 5 | 1.5 | 0.5 | 2 - steel | 13 | 0.75 | 0.25 | 2 - steel |
| 6 | 1.5 | 0.25 | 2 - steel | 14 | 0.75 | 0.5 | 2 - steel |
| 7 | 0.75 | 0.25 | 2 - steel | 15 | 1.5 | 0.25 | 2 - steel |
| 8 | 0.75 | 0.5 | 2 - steel | 16 | 1.5 | 0.5 | 2 - steel |

*Data pre-processing:* The missing data in the data set do not provide any information on the flank wear and will affect the model training. Here, we artificially delete these data points to form an all-supervised regression model. In this way, 15 out of 16 different conditions of data are retained because condition 6 has only one piece of data without labeled value. We use 14 pieces of flanks in 15 pieces as our training set and the last piece as the test set, respectively. Thus, we have totally trained 15 different models.

We define *X* as a matrix consisting of all-time interval signals in 14 training sets:

$X=\left[ \begin{matrix} X^{[1,1;1]} & \cdots& X^{[1,i;1]} & \cdots& X^{[1,9000;1]} \\ \vdots& & \vdots& & \vdots\\ X^{[k_{1},1;1]} & \cdots& X^{[k_{1},i;1]} & \cdots& X^{[k_{1},9000;1]} \\ \vdots& & \vdots& & \vdots\\ X^{[1,1;j]} & \cdots& X^{[1,i;j]} & \cdots& X^{[1,9000;j]} \\ \vdots& & \vdots& & \vdots\\ X^{[k_{j},1;j]} & \cdots& X^{[k_{j},i;j]} & \cdots& X^{\left[ k_{j},9000;j \right]} \\ \vdots& & \vdots& & \vdots\\ X^{[1,1;14]} & \cdots& X^{[1,i;14]} & \cdots& X^{\left[ 1,9000;14 \right]} \\ \vdots& & \vdots& & \vdots\\ X^{[k_{14},1;14]} & \cdots& X^{[k_{14},i;14]} & \cdots& X^{[k_{14},9000;14]} \end{matrix} \right]$

where the row represents the 9000 measurements according to one VB, $k_{j}$ denotes the maximum number of measurements of the *j_th_* condition, and $X^{[m,i;j]}$ *($1\leq m\leq k_{j}$)* represents the *i_th_* measured value in the *m_th_* measurements of the *j_th_* condition. Here we use *X^i^* to represent the *i_th_* column of matrix *X*. To ensure the uniformity of the data across all the conditions, we use the following formula to normalize the *i_th_* column of X, i.e., $X_{std}^{i}$:

$X_{std}^{i}=\frac{X^{i}-\mu^{i}1_{N}}{\sigma^{i}} ,$ (7)

where $\mu^{i}$ and $\sigma^{i}$ are the average and standard deviation values of $X^{i}$, and $1_{N}$ denotes all-ones vector of length *N*. After above standardisation for training set, we use the $\mu^{i}$ and $\sigma^{i}$ in training set to standardise the test set.

*Prediction results:* The proposed CNN model achieves a small mean square error (MSE) of ${10}^{-3}$ mm mostly in different test sets. We define $y_{m,real}$ as the measured VB of the *m_th_* measurements in $\mathrm{the}$ test data, and $y_{m,estimate}$ as the predicted values. In an arbitrary testing condition consisting of *N* pieces of time-course measurements according to *N* VBs, the MSE is given as follows:

$\mathrm{MSE}=\frac{1}{N}\sum_{m=1}^{N} ({y_{m,real}-y_{m,estimate})}^{2}.$ （8）

Other regression metrics such as RMSE, $R^{2}$ and MAE are given as follows:

R$\mathrm{MSE}=\sqrt{\frac{1}{n}\sum_{m=1}^{N} ({y_{m,real}-y_{m,estimate})}^{2}},$ （9）

$R^{2}=1-\frac{\sum_{m=1}^{N} \left( y_{m,real}-y_{m,estimate} \right)^{2}}{\sum_{m=1}^{N} \left( y_{m,real}-\bar{y} \right)^{2}},$ （10）

$\mathrm{MAE}=\frac{1}{N}\sum_{m=1}^{N} \left| y_{m,real}-y_{m,estimate} \right|,$ （11）

where $\bar{y}$ denotes an average of measured VBs in a predicted condition. The prediction results of condition 1, condition 3, condition 4, condition 5 and condition 8 are shown in Fig. S20. Detailed tuning parameters can refer to Fig. S28 and the prediction errors in each case are shown in Table S8.


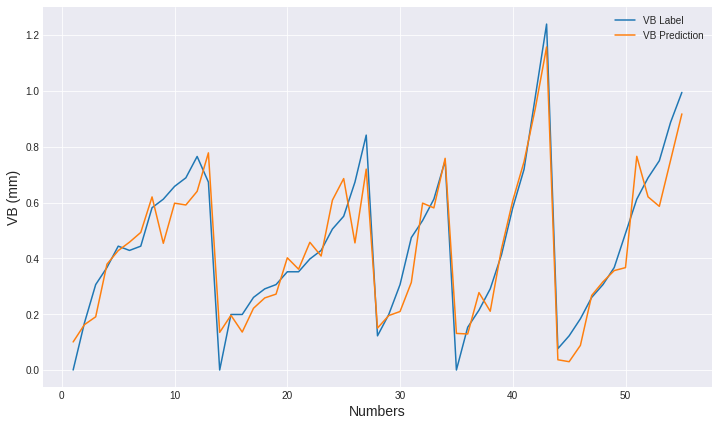


Fig. S27. NASA Tool wear regression of conditions 1, 3, 4, 5 and condition 8 respectively.


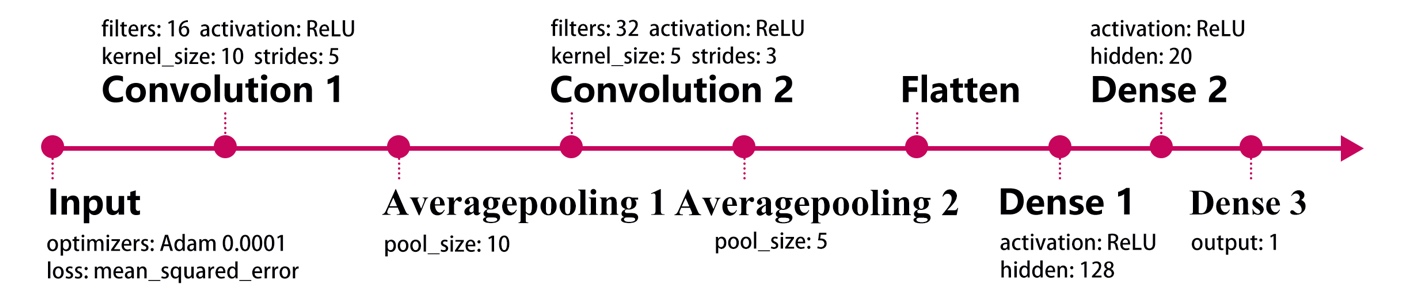


Fig. S28. CNN structure and parameters of the NASA tool wear value prediction task. The parameters of two inputs are the same.

Table S11: The results of NASA tool wear regression.

| Condition | MSE | MAE | R2 | RMSE |
| --- | --- | --- | --- | --- |
| 1 | 0.00718 | 0.07018 | 0.84353 | 0.09743 |
| 3 | 0.00883 | 0.07310 | 0.78886 | 0.08473 |
| 4 | 0.00583 | 0.05584 | 0.86914 | 0.09398 |
| 5 | 0.00428 | 0.05516 | 0.97112 | 0.07635 |
| 8 | 0.00949 | 0.08137 | 0.88987 | 0.06542 |
| Average | 0.00712 | 0.06713 | 0.87250 | 0.08358 |

# Case 9: NASA lithium-ion battery data for State of Health (SOH) estimation

*Problem formulations:* Lithium-ion batteries (LiBs) are the auxiliary or main power sources for many electronic systems, including medical devices, aerospace systems, smart phones, and electric vehicles [21]. Compared with conventional batteries such as lead-acid and nickel-cadmium, LiBs offer better lifespan, energy density and efficiency. The SOH reflects the condition (capacity) of a battery compared with its ideal condition. Battery management system (BMS) is proposed to manage the LiBs working safely and effectively, and SOH estimation is one of the essential functions among the BMS [22]. In practical terms, when the capacity of a battery is lower than 70-80% of initial value, battery failure is considered to occur. Battery failure during operation may cause reduced performance, profile loss, downtime and even catastrophic failure. Thus, an accurate SOH provides valuable information to the BMS.

*Introduction and properties of data set:* A benchmark data set for SOH estimation obtained by Prognostics Center of Excellence at NASA Ames Research Center [23] is investigated. Experiment test bed at NASA for the battery prognostics contains power supply, electrochemical impedance spectrometry (EIS), voltmeter, programmable DC electronic load as well as a PXI-based data acquisition system [24]. Three batteries (denoted by #5, #6, and #7) are tested with multiple cycles to accelerate battery aging through three operational profiles, namely charge, discharge and impedance, at room temperature. Properties of this data set are summarized as follows:

- Batteries run from their fresh rated capacity (2 Ah) until 30% fade condition (1.4 Ah).
- Measurement signals contain voltage, current, time, capacity, and temperature.
- Discharge processes of all the batteries are carried out at a constant current of 2 A. Voltage decreases from 4.2 V to 2.7 V, 2.5 V, and 2.2 V for #5, #6, and #7, respectively.
- Discharge voltage sequences and discharge capacity degradation trend are chosen as the input and label for CNN modeling, respectively.
- Our CNN framework is evaluated with a cross model validation, by randomly choosing two of the three batteries for the training of CNN model, while the obtained model is utilized to predict the SOH of the rest one. All the tuning parameters are the same in three models.

*Data pre-processing:* Initial discharge voltage 4.2 V is inserted into discharge voltage sequences to ensure the uniformity of the input sequence length for CNN modeling.

*Prediction results:* The regression results are shown in Fig. S29 and the corresponding prediction results are quantitatively summarized in Table S12. High average correlation coefficient ($R^{2}$) and small errors (MSE, MAE, and RMSE) indicate that the predicted value can be equivalent to the true capacity of the battery SOH. Tuning parameters, such as kernel size and max-pooling size, can refer to Fig. S30.


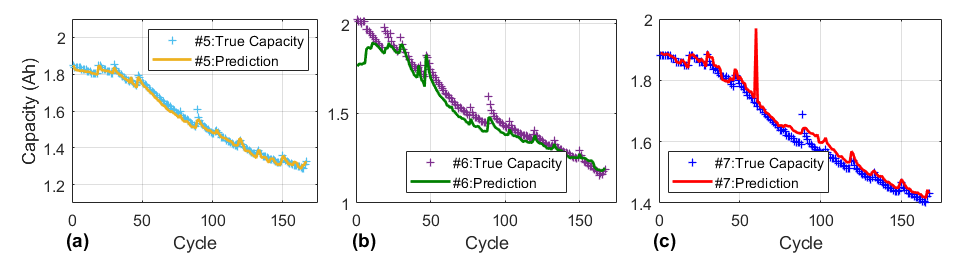


Fig. S29. SOH degradation curve predictions of (a) #5, (b) #6, and (c) #7 NASA Libs using a cross model validation approach.

Table S12: The predicted results of three batteries.

| Battery ID | MSE | MAE | R^2^ | RMSE |
| --- | --- | --- | --- | --- |
| # 5 | 3.46×10^-5^ | 0.00462 | 0.99593 | 0.00588 |
| # 6 | 0.00104 | 0.02204 | 0.91420 | 0.03232 |
| # 7 | 0.00018 | 0.00888 | 0.96990 | 0.01341 |
| **Average** | **4.20×10^-4^** | **0.01185** | **0.96001** | **0.01720** |


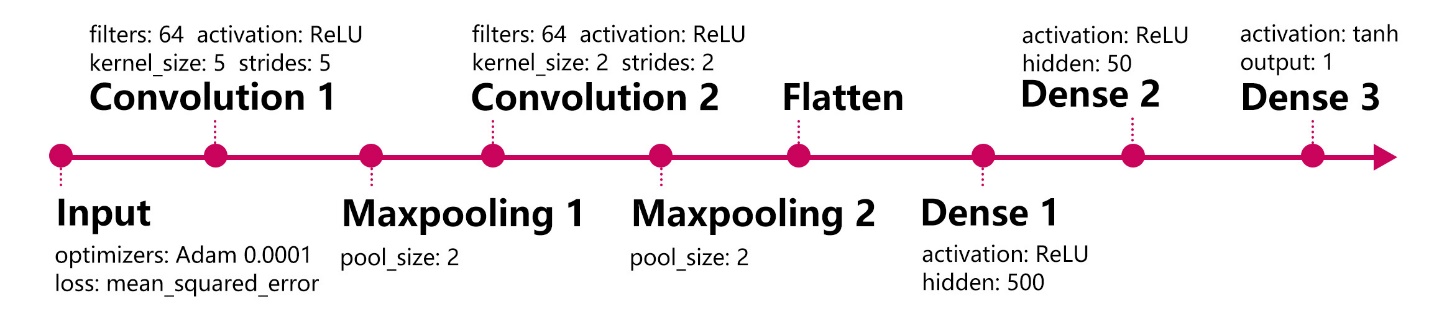


Fig. S30. Regression parameters in SOH prediction of NASA battery data.

*Comparison and discussions:* To illustrate the performance of our proposed framework, other state-of-the-art methods used for the same data set are employed for comparison. The results are given in Table S13. Our proposed CNN model achieves an average RMSE of 0.0172, which outperforms the previous works [25,26].

Table S13: The comparison results with other authors of NASA lithium-ion batteries.

|  | MSE (Average) | MAE (Average) | R^2^ (Average) | RMSE (Average) |
| --- | --- | --- | --- | --- |
| **Proposed model** | **4.20×10^-4^** | **0.01185** | **0.96001** | **0.0172** |
| Dong *et al.* [25] | - | - | - | 0.0264 |
| Zhou *et al.* [26] | - | - | - | 0.0292 |

# Case 10: CALCE lithium-ion battery data for State of Health (SOH) estimation

*Introduction and properties of data set:* With the same aim as *Case 9*, in this case, our CNN model is constructed for estimating the SOH of a CS2 type battery. The battery capacity data used in this case is provided by the Center of Advanced Life Cycle Engineering (CALCE), University of Maryland [27]. Under room temperature, CS2 cells undergo multiple charge-discharge cycles on an Arbin BT2000 battery testing system [28]. CS2 battery [29], charging/discharging cycle [30], and the Arbin testing system [31] are shown in Fig. S31. Properties of this data set are summarized as follows:

- 4 batteries (denoted by #35, #36, #37, and #38) are tested under a rated capacity of 1.1 Ah. All batteries experience the same discharge profile with a constant discharge current of 0.5 C. The voltage is discharged from 4.2 V to 2.7 V in each cycle.
- The data set of each battery consists of 16 types of parameters, including test time, cycle index, current, voltage, charging capacity, and discharging capacity.
- Discharge voltage sequences are measured as the input for CNN training.
- The discharge capacity (Ah) degradation trend is calculated as the label (health index) to predict the batteries’ SOHs.
- Due to the limitation of the available samples for training and testing, our CNN framework is evaluated with cross model validation. We randomly choose 3 of the 4 batteries for the training of CNN model to predict the SOH of the remaining one. All the tuning parameters are the same in four models.

Fig. S31. (a) CS2 battery with LiCoO2 cathode, weight=21.1g, and dimensions=5.4mm$\times$33.6mm$\times$50.6mm, (b) charging/discharging cycle, and (c) Arbin BT2000 battery testing system.

*Data pre-processing:* As the time intervals of two test points varies from 0 to 30 s, the acquired initial discharge voltage might be slightly smaller than 4.2 V. To ensure the uniformity of the input sequence length, 4.2 V is inserted as the first points of the discharge voltage sequences used for CNN modeling.

*Prediction results:* The prediction results of CALCE batteries are shown in Fig. S32. We can observe that our method performs excellent prediction performance compared with the true capacity curve, for all tested batteries. To further evaluate quantitative performance (see Table S14), we adopt MSE, MAE, $R^{2}$, and RMSE of SOH prediction to analyze the effectiveness of our proposed method. High average correlation ($R^{2}$=0.99540) and small average errors (MSE=2.39$\times{10}^{-4}$, MAE=0.00631, and RMSE=0.01365) are observed in four batteries. Detailed tuning parameters, such as kernel size and max-pooling size, can refer to Fig. S33.


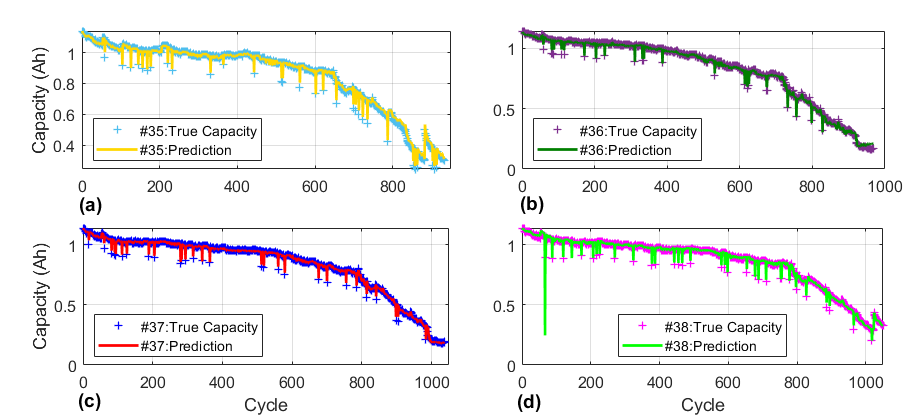


Fig. S32. Capacity degradation curve predictions of (a) #36, (b) #36, (c) #37, and (d) #38 CALCE LiBs using a cross model validation approach.

Table S14: The predicted results of CALCE batteries.

| Battery ID | MSE | MAE | R^2^ | RMSE |
| --- | --- | --- | --- | --- |
| # 35 | 0.00011 | 0.00726 | 0.99768 | 0.01064 |
| # 36 | 0.00012 | 0.00800 | 0.99829 | 0.01111 |
| # 37 | 4.49×10^-5^ | 0.00426 | 0.99922 | 0.00670 |
| # 38 | 0.00068 | 0.00572 | 0.98642 | 0.02613 |
| **Average** | **2.39×10^-4^** | **0.00631** | **0.99540** | **0.01365** |


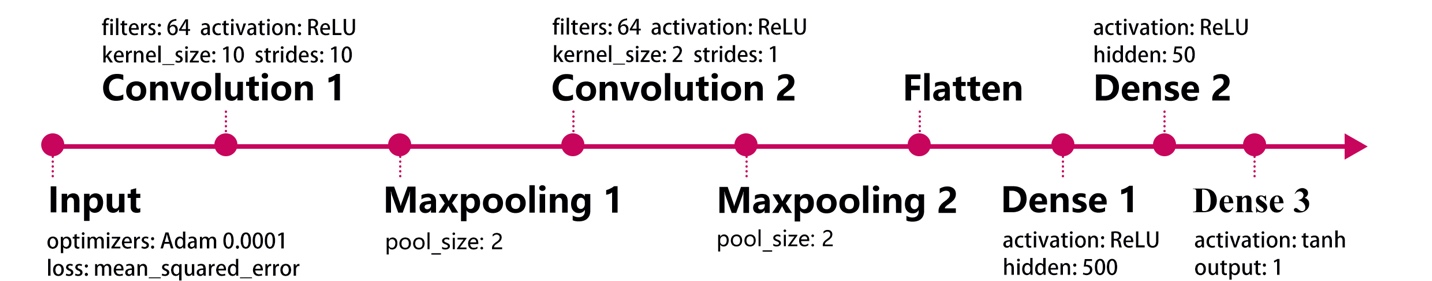


Fig. S33. Regression parameters in SOH prediction of CALCE battery data.

*Comparison and discussions:* To illustrate the performance of our proposed framework, other results that used the same data set are employed for comparison. The results are given in Table S15. Our proposed CNN model achieves an average RMSE of 0.01365, which outperforms the previous prediction performance.

Table S15: The comparison results with other authors of CALCE lithium-ion batteries.

|  | MSE(Average) | MAE(Average) | R^2^(Average) | RMSE(Average) |
| --- | --- | --- | --- | --- |
| **Proposed model** | **2.39×10^-4^** | **0.00631** | **0.99540** | **0.01365** |
| Huang *et al.* [32] | - | - | 0.97625 | 0.03687 |

# **References**

1. Shiroishi J, Y. Li SL, Danyluk S *et al.* Vibration analysis for Bearing outer race condition diagnostics. *J Brazilian Soc Mech Sci* 1999;**21**:484–92.

2. Saruhan H, Saridemir S, Çiçek A *et al.* Vibration analysis of rolling element bearings defects. *J Appl Res Technol* 2014;**12**:384–95.

3. Bearing Data Center. Case Western Reserve University Seeded Fault Test. http://csegroups.case.edu/bearingdatacenter/pages/download-data-file. (28 August 2018, date last accessed).

4. Shao H, Jiang H, Zhang X *et al.* Rolling bearing fault diagnosis using an optimization deep belief network. *Meas Sci Technol* 2015;**26**:115002.

5. Fawcett T. An introduction to ROC analysis. *Pattern Recognit Lett* 2006;**27**:861–74.

6. Longford NT. A fast scoring algorithm for maximum likelihood estimation in unbalanced mixed models with nested random effects. *Biometrika* 1987;**74**:817–27.

7. Shao H, Jiang H, Zhang H *et al.* Rolling bearing fault feature learning using improved convolutional deep belief network with compressed sensing. *Mech Syst Signal Process* 2018;**100**:743–65.

8. Samanta B, Al-Balushi KR, Al-Araimi SA. Artificial neural networks and support vector machines with genetic algorithm for bearing fault detection. *Eng Appl Artif Intell* 2003;**16**:657–65.

9. Yang BS, Han T, An JL. ART-KOHONEN neural network for fault diagnosis of rotating machinery. *Mech Syst Signal Process* 2004;**18**:645–57.

10. Sreejith B, Verma a. K, Srividya a. Fault diagnosis of rolling element bearing using time-domain features and neural networks. *2008 IEEE Reg 10 Third Int Conf Ind Inf Syst* 2008:1–6.

11. Chebil J, Noel G, Mesbah M *et al.* Wavelet decomposition for the detection and diagnosis of faults in rolling element bearings. *Jordan J Mech Ind Eng* 2009;**3**:260–7.

12. Vakharia V, Gupta VK, Kankar PK. A multiscale permutation entropy based approach to select wavelet for fault diagnosis of ball bearings. *JVC/Journal Vib Control* 2015;**21**:3123–31.

13. Liu X. Study on Knowledge -based Intelligent Fault Diagnosis of Hydraulic System. *TELKOMNIKA* 2012;**10**:2041–6.

14. *UCI Machine Learning Repository: Data Sets.* https://archive.ics.uci.edu/ml/datasets.html. (28 August 2018, date last accessed).

15. Helwig N, Pignanelli E, Schutze A. Condition monitoring of a complex hydraulic system using multivariate statistics. *Conference Record - IEEE Instrumentation and Measurement Technology Conference*. Pisa: IEEE, 2015, 210–5.

16. Mad Net. *Mad Data Set.* http://mad-net.org:8765/explore.html?t=0.597370213951085. (28 August 2018, date last accessed).

17. He Q, Guo Y, Wang X *et al.* Gearbox Fault Diagnosis Based on RB-SSD and MCKD（In Chinese）. *Zhongguo Jixie Gongcheng/China Mech Eng* 2017;**28**:1528–34.

18. Moldavska A, Welo T. The concept of sustainable manufacturing and its definitions: A content-analysis based literature review. *J Clean Prod* 2017;**166**:744–55.

19. 2016 World Machine Tool Survey : Gardner Intelligence. ﻿https://www.gardnerintelligence.com/articles/2016-world-machine-tool-survey. (28 August 2018, date last accessed).

20. A. Agogino and K. Goebel (2007). BEST lab, UC Berkeley. "Milling Data Set ", NASA Ames Prognostics Data Repository (http://ti.arc.nasa.gov/project/prognostic-data-repository), NASA Ames Research Center, Moffett Field, CA.

21. Omariba Z, Zhang L, Sun D. Review on Health Management System for Lithium-Ion Batteries of Electric Vehicles. *Electronics* 2018;**7**:1–26.

22. Zheng Y, Ouyang M, Han X *et al.* Investigating the error sources of the online state of charge estimation methods for lithium-ion batteries in electric vehicles. *J Power Sources* 2018;**377**:161–88.

23. B. Saha and K. Goebel (2007). “Battery Data Set”, NASA Ames Prognostics Data Repository (http://ti.arc.nasa.gov/project/prognostic-data-repository), NASA Ames Research Center, Moffett Field, CA.

24. Saha B, Goebel K. Modeling Li-ion battery capacity depletion in a particle filtering framework. *Proceedings of the Annual Conference of the Prognostics and Health Management Society*. 2009, 2909–2924.

25. Dong G, Chen Z, Wei J *et al.* Battery health prognosis using brownian motion modeling and particle filtering. *IEEE Trans Ind Electron* 2018;**65**:8646–55.

26. Zhou D, Xue L, Song Y *et al.* On-Line Remaining Useful Life Prediction of Lithium-Ion Batteries Based on the Optimized Gray Model GM(1,1). *Batteries* 2017;**3**:1–17.

27. He W, Williard N, Osterman M *et al.* Prognostics of lithium-ion batteries based on Dempster-Shafer theory and the Bayesian Monte Carlo method. *J Power Sources* 2011;**196**:10314–21.

28. Battery Cell Test Equipment. *Arbin Instruments.* ﻿https://www.arbin.com/products/battery-testing/cell-testing. (28 August 2018, date last accessed).

29. He W, Williard N, Osterman M *et al.* Prognostics of lithium-ion batteries based on Dempster-Shafer theory and the Bayesian Monte Carlo method. *J Power Sources* 2011;**196**:10314–21.

30. ARBIN BT2000. *Battery Test Equipment.* ﻿http://slategrey.pt/wp-content/uploads/2016/06/BT-2000-Multi-Channel-Battery-Testing-System-da-ARBIN.pdf. (28 August 2018, date last accessed).

31. Software. *Arbin Instruments.* ﻿https://www.arbin.com/software. (28 August 2018, date last accessed).

32. Huang SC, Tseng KH, Liang JW *et al.* An online SOC and SOH estimation model for lithium-ion batteries. *Energies* 2017;**10**:1–18.
